# Detecting the tipping points in a three-state model of complex diseases by temporal differential networks

**DOI:** 10.1186/s12967-017-1320-7

**Published:** 2017-10-26

**Authors:** Pei Chen, Yongjun Li, Xiaoping Liu, Rui Liu, Luonan Chen

**Affiliations:** 1 School of Computer Science and Engineering, South China University of Technology, Guangzhou, 510640 China; 2 School of Mathematics and Statistics, Shandong University at Weihai, Weihai, 264209 China; 3 Collaborative Research Center for Innovative Mathematical Modelling, University of Tokyo, Tokyo, 153-8505 Japan; 4 School of Mathematics, South China University of Technology, Guangzhou, 510640 China; 5 Key Laboratory of Systems Biology, Shanghai Institutes for Biological Sciences, Chinese Academy of Sciences, Shanghai, 200031 China

**Keywords:** Critical transition, Pre-disease state, Differential network, Dynamical network biomarker, Hidden Markov model, Tipping point

## Abstract

**Background:**

The progression of complex diseases, such as diabetes and cancer, is generally a nonlinear process with three stages, i.e., normal state, pre-disease state, and disease state, where the pre-disease state is a critical state or tipping point immediately preceding the disease state. Traditional biomarkers aim to identify a disease state by exploiting the information of differential expressions for the observed molecules, but may fail to detect a pre-disease state because there are generally little significant differences between the normal and pre-disease states. Thus, it is challenging to signal the pre-disease state, which actually implies the disease prediction.

**Methods:**

In this work, by exploiting the information of differential associations among the observed molecules between the normal and pre-disease states, we propose a temporal differential network based computational method to accurately signal the pre-disease state or predict the occurrence of severe disease. The theoretical foundation of this work is the quantification of the critical state using dynamical network biomarkers.

**Results:**

Considering that there is one stationary Markov process before reaching the tipping point, a novel index, inconsistency score (*I*-score), is proposed to quantitatively measure the change of the stationary processes from the normal state so as to detect the onset of pre-disease state. In other words, a drastic increase of *I*-score implies the high inconsistency with the preceding stable state and thus signals the upcoming critical transition. This approach is applied to the simulated and real datasets of three diseases, which demonstrates the effectiveness of our method for predicting the deterioration into disease states. Both functional analysis and pathway enrichment also validate the computational results from the perspectives of both molecules and networks.

**Conclusions:**

At the molecular network level, this method provides a computational way of unravelling the underlying mechanism of the dynamical progression when a biological system is near the tipping point, and thus detecting the early-warning signal of the imminent critical transition, which may help to achieve timely intervention. Moreover, the rewiring of differential networks effectively extracts discriminatively interpretable features, and systematically demonstrates the dynamical change of a biological system.

**Electronic supplementary material:**

The online version of this article (doi:10.1186/s12967-017-1320-7) contains supplementary material, which is available to authorized users.

## Background

**Fig. 1:**
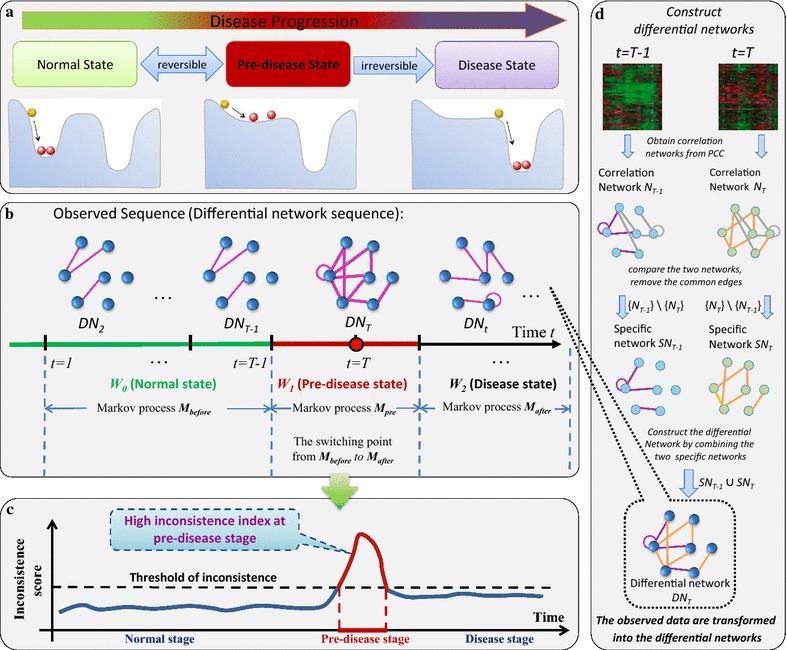
Outline for identifying the pre-disease state by using the differential-network-based HMM. **a** Disease progression can be divided into three states, i.e., the normal state with high resilience, the pre-disease state with low resilience, and the disease state with possible high resilience. **b** First, we constructed the differential network sequence *O*_*T* − 1_ = {*DN*_2_, *DN*_3_, …, *DN*_*T* − 1_} based on the observed molecular data. Then, we trained the HMM $\varTheta_{T - 1} = (A_{T - 1} ,\;B_{T - 1} ,\;\pi_{T - 1} )$ representing the normal state, which, in view of stable dynamics, was modelled as the stationary Markov process *M*_*before*_, while on the other hand, the pre-disease state was defined as a Markov process *M*_*pre*_. Thus, based on the dynamical difference of the two Markov processes, detecting the pre-disease state during the disease progression is equivalent to identifying the switching point between these two distinct Markov processes. Second, for each candidate time point, we calculated the probability *P* of being the switching point based on the HMM $\varTheta_{T - 1} (O_{T - 1} )$. **c** The abrupt increase of *P* indicated that a candidate point *t* = *T* was the switching point of $\varTheta_{T - 1} (O_{T - 1} )$ with high probability. **d** The differential network was obtained based on three steps at each time point or period. We first constructed the correlation network at each time point. Comparing the correlation networks from adjacent time points, this generated a specific network, which included the specific edges for each time point. Then it followed the differential network by combining the specific networks from adjacent time points

The deterioration process of many complex diseases is not always smooth but occasionally abrupt, that is, there are critical states just before such drastic changes/transitions during disease progression, which may signal the emergence of disease states [1]. For example, in some chronic diseases such as cancer [2–4], serious deterioration may occur suddenly within a short period of progression, while before such catastrophic transition the disease (e.g., the process of chronic inflammation) may progress gradually and steadily for years or even decades of a long incubation period. A nonlinear disease progression, a continuum of progressive changes occurring in micro- and macro-environment of a certain organ or the whole organism, is undoubtedly complex. Regardless of specific differences in either biological processes or observed symptoms among diseases, the progression of illness is divided into three stages or states from computational point of view (Fig. 1a), i.e., a normal state, a pre-disease state, and a disease state, where the pre-disease state is a critical state just before disease appearance [5–7]. In other words, during the course of illness there is an unexpected transition from a relatively healthy/normal stage via a critical/pre-disease stage to a severe disease stage [8–13]. Thus, it is crucial to signal such pre-disease states for many complex diseases, before their progressions transit to the irreversible disease states. This could prevent or at least allow for preparation for the catastrophic events.

Although great efforts have been devoted to the diagnosis of complex diseases, it is still a challenging task to identify the onset of pre-disease state or predict the disease state by finding accurate and robust biomarkers specific to respective diseases. Traditional biomarkers, including molecular- or module-markers, are employed to distinguish disease samples from normal samples or identify the disease state by exploiting the information of the differential expressions for the observed molecules between the normal and disease states, rather than diagnosing the pre-disease state which generally has no significant static differences from the normal states. This is also the reason that the clinical judgments through traditional biomarkers may fail to signal a pre-disease state. Besides, in conventional identification, biomarkers are usually selected by identifying single differential biomolecules. However, given the functional interdependencies among molecular components in a human cell, a disease phenotype is rarely a consequence of an abnormality in single biomolecules, but reflects the change of complex interaction network associated with genes, proteins and metabolites [14]. Therefore, the understanding of the dynamical change in a biomolecule interaction network is essential in comprehensively studying the progression of complex diseases and thus better detecting the related warning signals. By exploring the information of differential associations among the observed molecules between the normal and pre-disease states, a new concept of dynamical network biomarker (DNB) [3, 15] with its statistical conditions was proposed to detect early-warning signals before disease onset at the molecular network level. Having been applied to real biological and clinical data, the DNB strategy and its follow-up modifications had signalled the pre-disease state of several diseases [7, 16–21]. Our DNB model has also been employed by many groups, successfully detecting the tipping points of cell fate decision [22], cellular differentiation [23] and further investigating the immune checkpoint blockade [24]. However, the classic DNB method relies on accurate high-throughput data with small noise to correctly choose the dominant biomolecules, which is generally not available for many cases.

In this work, from the viewpoint of differential networks, we propose a computational approach based on an unsupervised hidden Markov model (HMM) to automatically detect the early-warning signal of the tipping/critical point during disease progression. Our previous theoretical derivations indicated that the pre-disease and normal states are statically similar in terms of molecular expressions, but are dynamically different [15, 16, 25]. To exploit such features on their dynamical differences, we first construct a sequence of differential networks on the basis of time-course data, to integrate biomolecules with temporally distinct features including genes with differential expression variance and interactions between gene-pairs with differential expression covariance. Describing the temporal change between two networks respectively acquired from adjacent sampling time points, this differential information and the altered network structure derived therefrom systematically show the dynamical change of a biological system from the perspective of differential network. Then, to better quantify these dynamical differences and accurately identify the critical period, we consider that the progression of a network system during each stage can be represented by a specific Markov process because of their dynamical nature. Then, the whole phase transition dynamics of a biological system is represented as three different Markov processes [25]. The pre-disease state can be regarded as a time-varying Markov process in view of its high sensitivity to even small perturbations or parameter changes during this stage (Fig. 1b). Thus, signalling the pre-disease state is equivalent to detecting the switching point of the first stationary Markov process (the normal state). Utilizing a temporal series of differential networks generated from time-course data (Fig. 1d), we proposed a computational approach for estimating the possibility distribution of the switching dynamics at each candidate sampling point. Specifically, by exploring the differential associations (or differential network) in dynamics (Fig. 1b), we propose an quantitative index called inconsistency score (*I*-score) as a specific identifier for signalling the impending tipping point, i.e., the rapid rise of *I*-score indicates the emergence of the pre-disease state, while the *I*-score develop smoothly with little significant fluctuation in either a normal state or disease state (Fig. 1c). To demonstrate the effectiveness, our approach is applied to a numerical experiment and three real datasets of diseases, including the microarray dataset of acute lung injury triggered by inhalation of carbonyl chloride (GSE2565), the microarray dataset of stimulating-stress production caused by acute corneal trauma (GSE1393), and genomic data of heregulin-induced breast cancer (GSE13009). The onset of pre-disease state were successfully signalled for both numerical simulation and the three real genomic datasets.

## Methods

We first demonstrate the theoretical background, i.e., the dynamical behaviour of a complex system when approaching to the tipping point or bifurcation point. Then the detailed algorithm used in this study is provided.

### Three Markov processes and related statistic conditions

The progressions of most diseases are generally divided into the following three states/stages: normal state, pre-disease state and disease state (Fig. 1a). Both the normal state and the disease state are stable or stationary with high resilience or little fluctuations, and are robust to parameter changes. For these two states, it is not easy to trigger a critical transition towards an alternative state through external perturbations [21]. Thus, these two states are modelled as two stationary Markov processes (Fig. 1b). The pre-disease state is a state defined as the limit of the normal state just before the critical phase transition. Different from the above two stable states, the pre-disease state is a time-varying state with low resilience or strong fluctuations, and sensitive to even a small parameter change [21]. However, it may reverse back to the normal state if appropriately treated, which is in contrast to the irreversible disease state. Statically, there are little state change or significant differences such as consistently high/low expressions in some single biomolecules between the normal and pre-disease states [26]. Taking the dynamically unstable nature of the pre-disease state into account, it is regarded as a time-varying Markov process with strong fluctuating dynamics. For a specific disease, the pre-disease state presents the critical stage between a relatively “healthy” stage (the normal state) and a seriously ill stage (the disease state) in which it is difficult for a system to recover to the normal state even intensive interferences involved. Thus, it is of great importance to signal the pre-disease state in order to prevent catastrophic deterioration by performing timely intervention.

The DNB strategy suggests that a dominant group of molecules or DNB module appears at the pre-disease state. In such a group, the molecules together provides an early-warning signal when the biological system is in the vicinity of the tipping point [1]. Specifically, we theoretically proved that when a biological system from a normal state approaches the tipping point of a critical transition, the DNB module appear and simultaneously satisfy the following three statistic conditions [3, 15].

(i) The standard deviation of each molecule in the module drastically increases.
(ii) The correlation between molecules both within the module (i.e., intra-class correlation) sharply increases.
(iii) The correlation between molecules inside and outside the module (i.e., inter-class correlation) rapidly decreases.

The derivation of the three generic conditions is presented in Additional file 1: A1 and A2. It also should be noted that the three-state model of complex diseases is not always generalizable. Actually, when there is no abrupt deterioration phenotype during the disease progression, or a stage-wise division is not applicable, such model is improper to characterize the biological processes of diseases.

### Three Markov processes and related statistic conditions

Most biological molecules perform their functions through interactions with other biomolecules, which are either in a functional module or across modules. This inter- and intra-module interconnectivity suggests that the impact of a specific genetic abnormality not only affects the activity of the gene product that carries it, but may extend along the links of a network composed by biomolecules and change the activity of other gene products [27]. Therefore, an understanding of a biomolecule’s interaction network context is essential in determining the phenotypic impact of defects that affect it.

To study the dynamical evolution of a network system during the switch of a stationary Markov process into another time-varying Markov process, we employed a differential network that integrates differential associations/edges, including differential expression variances and differential expression correlations from adjacent sampling time points (Fig. 1d), that is, to quantify the statistical significance of each differential edge (differential Pearson’s correlation coefficient (PCC) value) in a differential network [28].

The difference between this differential network and existing methods lies in that we study the differential associations/correlations of genes/proteins rather than differential expressions of genes/proteins. In particular, we assume that the dysfunction or network rewiring of those associations during disease progression is possibly related to the relevant disease processes. Using gene or protein expression to quantitatively describe the dynamic associations between biomolecules, we can identify those significantly changed associations (i.e., gene pairs rather than individual biomolecules) between two biomolecules. These are denoted as differential associations hereafter and are presumed to be highly related to the dysfunction of regulation network of interest. The temporal-ordering differential network was obtained by the following three steps (Fig. 1d).

#### Building the correlation network

By mapping the correlation to an existing functional network (i.e., an integrated STRING network), the correlation network *N*_*T*_ was constructed at each sampling time point *t* = *T* (Fig. 1). Each edge connecting two nodes represents the correlation/association between two biomolecules, while each edge connecting only one node represents the self-regulation or variation of the biomolecule. Subsequently, there is a parameter α such that only those edges with high Pearson’s correlation coefficients (PCC) $(\left| {PCC} \right|\; \ge \;\alpha )$ or high standard deviation (SD) ($\frac{{SD - { \hbox{min} }\left( {SD} \right)}}{{\hbox{max} \left( {SD} \right) - mean\left( {SD} \right)}} \ge \alpha$) were reserved, where *α* is a to-be-determined parameter based on specific real data, that is, α is set such that there are few differential edges (i.e., significantly changed associations) in the initial differential networks during the normal stage, thus highlighting the pre-disease stage when many edges arise. The estimation of *α* is discussed in Additional file 1: B.

#### Building the specific network

Comparing the correlation networks *N*_*T*_ and *N*_*T* − *1*_, and the edges which differed between *N*_*T*_ and *N*_*T* − *1*_ were then identified and employed to illustrate the dynamic changes in associations between the adjacent time points *T* and *T* − *1*. Then we obtained the specific networks $SN_{T} = \left\{ {N_{T} } \right\}\backslash \left\{ {N_{T - 1} } \right\}$ and $SN_{T - 1} = \left\{ {N_{T - 1} } \right\}\backslash \left\{ {N_{T} } \right\}$ respectively by removing the common edges (see Fig. 1d), where the common edges are presented in both correlation networks *N*_*T*_ and *N*_*T* − *1*_ and thus were regarded as interactions without significant change in dynamics.

#### Building the differential network

Combining the two specific networks *SN*_*T*_ and *SN*_*T* − *1*_, i.e., {*SN*_*T*_} ∪ {*SN*_*T* − 1_}, we obtained the differential network *DN*_*T*_ at time point *T*, in which the edge connecting two nodes records temporal differential correlation, and the edge connecting only one node records temporal differential variance.

We thus transformed the observed sequence (consecutive time-series data) {*o*_1_, *o*_2_, *o*_3_, …, *o*_*T*_, …} into a temporal-ordering differential network sequence {*DN*_2_, *DN*_3_, …, *DN*_*T* − 1_, *DN*_*T*_…} (Fig. 1b). Without abusing notation, we also denote the differential network series up to time *T* as $O_{T} = \left\{ {o_{1} ,o_{2} ,o_{3} , \ldots ,o_{T - 1} , o_{T} } \right\} = \left\{ {DN_{2} ,DN_{3} , \ldots ,DN_{T - 1} ,DN_{T} } \right\}$. Each edge in the differential network *DN*_*T*_ represents the differential association in dynamics, i.e., distinct edges between *N*_*T*_ and its preceding correlation network *N*_*T* − 1_. The disease-related genes are those that are common in the differential network sequence $DN_{2} , DN_{3} , \ldots ,DN_{T}$. It is very possible that the genes included in these differential networks play important roles in progression from the normal state to the pre-disease state. An intuitive view of the differential network is that there are few differential edges when the differential network is constructed during either a normal or a disease stage, since two adjacent specific networks are expected to possess similar structures in view of the high stability nature of the system during these stages. On the other hand, since two adjacent specific networks should be quite different due to the time-varying and fluctuating dynamics of the system, many differential edges are expected to arise abruptly in a differential network when the system steps into a pre-disease stage. A sequence of such differential networks can portray time-dependent alterations of the system, and thus monitor the change at different stages of disease progression. In order to explore and quantify such a dynamical feature, we propose the scheme as follows.

### Measuring the probability of being the end point of a stationary Markov process

According to the discussion above, the progression of a network system through a normal state is described by a stationary Markov process. Therefore, detecting the outset of a pre-disease state is equivalent to identifying the end of this stationary Markov process, or the switching point from a stationary Markov process to a time-varying Markov process. It is supposed that each time point $t = T (T > 2)$ is a candidate transition point, or equivalently, the switching point of a stationary Markov process. Then we need to determine whether the observable samples or differential network derived at this candidate point is not coincident with that in the preceding stationary Markov process. Therefore, an index *I*-score is proposed for measuring the probability of how likely a candidate time point is the ending point of a stationary Markov process. The approach contains the following two steps. First, utilizing an observable network sequence *O*_*T* − 1_ = {*DN*_2_, *DN*_3_, …, *DN*_*T* − 1_}, i.e., *T* − 2 temporal-ordering differential networks based on samples from the preceding time points *t* = 1, 2,…, *T* − 1, we trained and obtained a hidden Markov model (HMM) $\varTheta_{T - 1} \left( {O_{T - 1} } \right) = \left( {A,B,\pi } \right)$, with the subscript *T* − 1 of $\varTheta$ denoting that $\varTheta$ is constructed from the training samples/networks up to *t* = *T* − 1; parameter *A* represents a state transition matrix; *B* stands for an observation matrix; and *π* denotes the initial probabilities. The training of $\varTheta$ follows the Baum-Welch procedures, an unsupervised learning method, which is presented in Additional file 1: A3. Second, we test if a candidate point is belong to the switching period, by calculating the *I*-score of the form:

$$I\left( T \right) = P_{T} (s_{T} = S_{pre} | s_{T - 1} = S_{normal} , \ldots , s_{1} = S_{normal} ;\varTheta_{T - 1} ,O_{T} ), \left( {T \ge 2} \right)$$

where $\varTheta_{T - 1} (O_{T - 1} )\; = \;(A,\,B,\,\pi )$ is the trained HMM. *O*_*T*_ = {*DN*_2_, *DN*_3_, …, *DN*_*T*_} is a sequence of temporal-ordering differential networks. {*s*_1_, *s*_2_, …, *s*_*T* − 1_, *s*_*T*_} denotes a state sequence where *s*_*i*_ is the system state at the *i*-th time point. *S*_*normal*_ and *S*_*pre*_ are both hidden (unobserved) states. *S*_*normal*_ stands for the normal state, and *S*_*pre*_ represents for the pre-disease state. On one hand, if the differential network {*DN*_*T*_} is derived in the normal stage, or equivalently the testing point *t* = *T*, at which *DN*_*T*_ is built, is still within the stationary Markov process characterized by HMM $\varTheta_{T - 1}$, then the *I*-score *I*(*T*) is supposed to be identical or similar to *I*(*T* − 1) (Fig. 1c). Thus the *I*-score curve progresses steadily and remains low value if the system is in the normal state. On the other hand, once the observation {*DN*_*T*_} is from the pre-disease state, or the system transits into a time-varying Markov process at testing time point *t* = *T*, *I*(*T*) rises rapidly, suggesting that the observation *o*_*T*_ = {*DN*_*T*_} is highly inconsistent with the HMM $\varTheta_{T - 1}$ constructed from the prior information, i.e., the differential network series {*DN*_2_, *DN*_3_, …, *DN*_*T* − 1_}. Clearly the inconsistent observation or distinct differential networks only appear during the switching period between the normal stage described by a stationary Markov process and the pre-disease stage described by a time-varying Markov process. Therefore, the boost of *I*(*T*) signals the onset of the pre-disease stage, or the impending of catastrophic transition. The specific algorithm for calculating *I*-score is presented in Additional file 1: A3.

### Data processing

We applied the *I*-score scheme to three time-course datasets (GSE2565, GSE1393 and GSE13009) downloaded from the NCBI GEO database (http://www.ncbi.nlm.nih.gov/geo). For all these genomic data, we discarded the probes without corresponding NCBI Entrez gene symbol. For each gene mapped by multiple probes, the average value was employed as the gene expression. The procedure of building a molecular interaction network was as follows. First, the biomolecular association networks for *Homo sapiens* and *Mus musculus* were downloaded from several public databases, e.g., protein–protein interactions from STRING (http://string-db.org), and transcriptional regulations from TRED (rulai.cshl.edu/cgi-bin/TRED/tred.cgi?process = home). We integrated these linkage information together without redundancy into a whole molecular interaction network including 65,625 functional linkages in 11,451 molecules for *Homo sapiens*, and 37,950 linkages in 6683 molecules for *Mus musculus*. Second, the genes from each microarray dataset were mapped to the integrated network to extract the related linkages. The molecular network was used for the initial construction of correlation networks and consequent analysis. Finally, our main results were visualized by Cytoscape (http://www.cytoscape.org) in the post-processing step. The functional analysis including gene ontology and pathway enrichment were based on GO database (http://www.geneontology.org/page/go-database) and KEGG mapper tool (http://www.genome.jp/kegg/tool/map\_pathway2.html).

For real datasets, to reduce computational complexity, the differential network was partitioned into many local networks. Each local network contained a centre node (a differentially-expressed gene) and all of its first-order neighbours based on the network structure, such as a mapped STRING network. The *I*-scores for the local networks were then calculated one by one, thus generating a weighted average score. Specifically, suppose there are *k* subnetworks partitioned from a differential network,

$$I = \frac{{n_{1} I_{1} + n_{2} I_{2} + \cdots + n_{k} I_{k} }}{{n_{1} + n_{2} + \cdots + n_{k} }}$$

where *n*_*i*_ denotes the number of nodes in the *i*-th local network and *I*_*i*_ stands for the local *I*-score of this subnetwork.

In Additional file 1: C, data description and processing procedures were presented and discussed in detail.

## Results

### Validation of numerical experiments

In order to validate the proposed computational method and *I*-score, we employed a theoretical model of an eight-node regulatory network (Additional file 1: Figure S1) to illustrate the identification of a critical stage when the system approaches a tipping point. Such model of regulatory network which is of Michaelis–Menten form, is usually employed for studying genetic regulatory activities such as transcription, translation, diffusion, and translocation processes [29]. Detailed description of the network characterized by a set of eight stochastic differential equations, was provided in Additional file 1B. Based on a sequence of 23 sets of parameters, a dataset was generated for numerical simulation from the network.

**Fig. 2:**
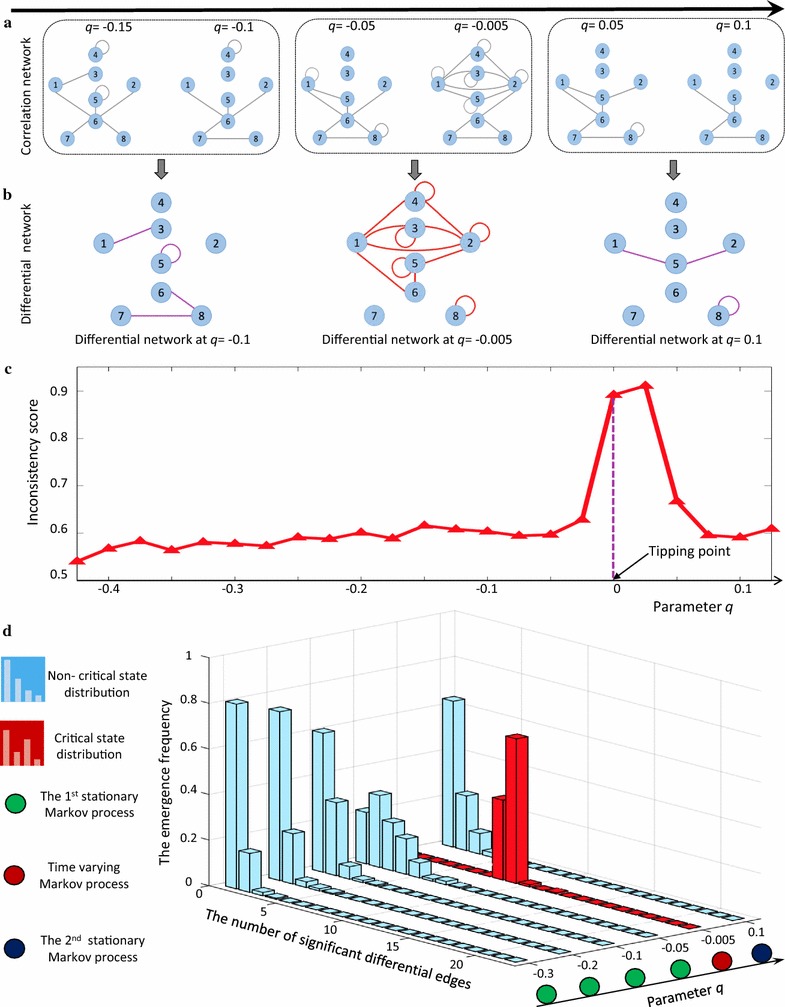
The validation of $\varvec{I}$-score through a numerical experiment. To validate our method, the *I*-score scheme was performed on a simulated dataset from an eight-node network, whose detailed description is in Additional file 1: B. **a** The specific eight-node correlation networks respectively constructed along the sequence of parameter *q*. Among the 6 consequent networks, the first three (*q* = − 0.15, *q* = − 0.1 and *q* = 0.1) are in the normal state and no significant difference among them; the fourth (*q* = − 0.005) represents the transition state; and the last two (*q* = 0.05 and *q* = 0.1) locate in the disease state. **b** The construction of 3 differential networks based on adjacent specific networks. In each differential network, the edge connecting two nodes records temporal differential correlation, and the edge connecting only one node records temporal differential variance. **c** The *I*-scores of the network system. A boost of *I*-score signals the tipping point at *q* = 0, which agrees with the fact that the system undergoes a bifurcation at *q* = 0. **d** The distribution of the occurrence frequency of differential edges in the differential network. When parameter *q* is far from the critical value *q* = 0 (*q* = − 0.15, *q* = − 0.1), there are few differential edges (in statistical sense). However, when *q* approaches the bifurcation value *q* = 0 (*q* = − 0.005), the distribution changes considerably, i.e., the ratio of 13-differential-edges increases significantly

In Fig. 2a and b, we demonstrated the generation of differential network based on time-course data. There were a sequence of specific correlation networks constructed (Fig. 2a), based on which consequent differential networks were built (Fig. 2b). Taking the differential networks as observable samples, we carried out the numerical experiment. It can be seen that a sharp increase of the *I*-score, i.e., probability of being a critical transition point, indicated the coming tipping point at a bifurcation parameter value *q* = 0 (Fig. 2b).

To exhibit the distinct dynamics of the system between the normal state and the pre-disease state, we illustrate the underlying mechanisms of the *I*-score in Fig. 2c, that is, the occurrence-frequency of differential edges changes along the progression of the system. It is clear that when the system is far away from the tipping point, few edges are in a differential network, while when the system approaches the tipping point, considerably more differential edges are expected to appear in a differential network. Through this numerical experiment, it is clear that our computational approach is model-free and works even we do not know which variable or subnetwork is important in detecting the early-warning signal. Thus *I*-score and differential networks are capable of exploiting the high-dimensional information and thus distinguishing pre-disease samples from normal samples. The numerical experiment validates that the *I*-score is reliable and accurate in signalling the critical/pre-disease stage.

We present the simulation and calculation details in Additional file 1: B.

### Predicting critical transitions in real datasets

We further identified the pre-disease state through *I*-score in three real time-course datasets, i.e., the gene expression profiling dataset (GSE2565) generated from a mouse experiment, in which pulmonary edema was triggered by inhalation of carbonyl chloride [30]; the microarray dataset (GSE1393) of acute corneal trauma after a chemical burn with silver nitrate was also obtained from a mouse experiment, which is carried out for examining the effect of stimulation stress produced by chemical burn, in comparison with the non-stimulated control animals [31]; and the genomic dataset (GSE13009) derived from an study on human breast cancer cells, in which heregulin induced sustained signal activity in MCF-7 cells and triggered an irreversible cell phenotype change to differentiation [32]. The application on the lung injury dataset is set as a concrete demonstration. A stepwise algorithm is also presented in Additional file 1: C.

**Fig. 3:**
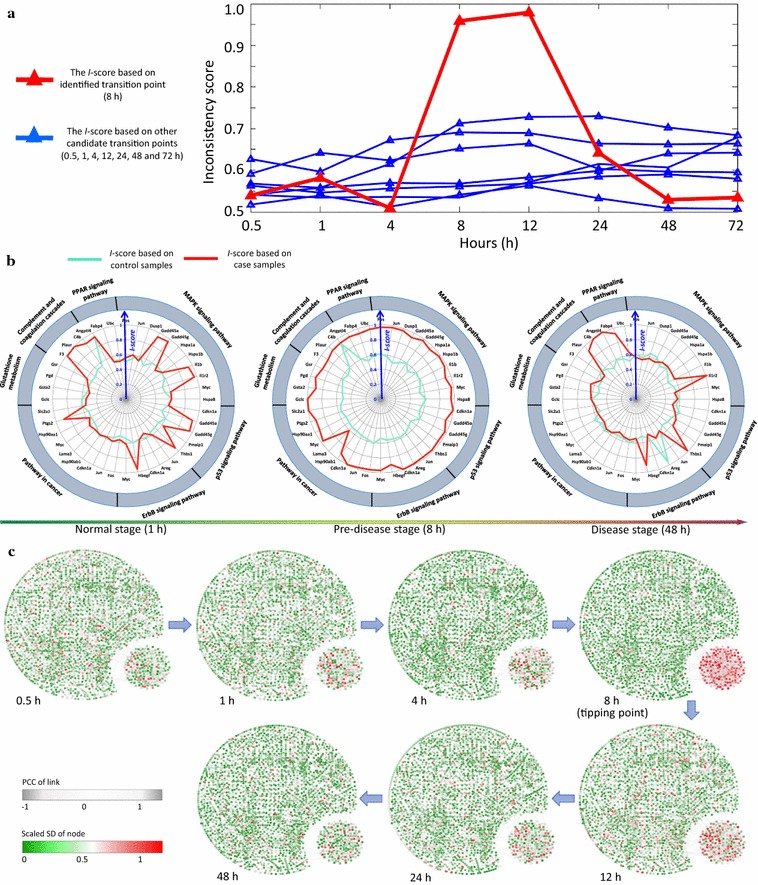
Application of $\textit{I}$-score on the microarray data of acute lung. **a** *I*-score curves based on the differential networks respectively constructed at each candidate critical time point. The red curve represents the *I*-score calculated from the testing differential network obtained at 8 h, and the seven blue curves are those derived from other candidate time points. The most significant signal appears at 8-hour point, which agrees with the experimental observation. **b** Radar plots present the dynamical change in *I*-scores of some local differential networks, which are labelled by their centre genes that enriched in pathways indicated outside. The red curve represents the *I*-score from case samples while the green curve is from the control data. At the pre-disease stage, the inconsistence is significant. **c** The dynamical evolution of the whole molecular network is shown respectively at 0.5, 1, 4, 8, 12, 24, and 48 h. The networks were constructed through a mapped whole mouse network. Node colour represents the fluctuation of expression, and the thickness of links represents the correlation between each pair of nodes. In the lower right corner of each network, there is a group of 189 genes with top 10% most significant *I*-scores’ change, which together show wild fluctuation in their expressions around 8 h

In the experiment of phosgene-induced mice lung injury (GSE2565), two groups of CD-1 male mice, each comprising six mice, were exposed to either air (as the control group) or phosgene (as the case group). Lung tissues were collected and processed from the two groups of mice at nine sampling points, i.e., 0, 0.5, 1, 4, 8, 12, 24, 48, and 72 h after exposure, based on which two series of gene expression data were produced. By comparing the gene expression levels between phosgene- and air-exposed mice, the experiments studied the oxidative detriments on lung tissue caused by phosgene exposure [30]. Identifying the critical point follows a procedural scheme. The differential network was constructed at each sampling point on the basis of differential-expression genes screened by the *P* value from a paired Student’s t test between case and control samples. According to the *I*-score scheme, except for the first sampling time point (0 h) which is always assumed as in a normal stage, we take each time point as a candidate critical point, that is, the candidate ending point of the stationary Markov process representing for the normal stage. The *I*-score was then calculated based on the testing differential network derived at each candidate critical point (Fig. 3a). Among the eight *I*-score curves in Fig. 3a, the red curve represents the inconsistency probability for testing the candidate time point at 8 h, and the seven blue ones are for testing other candidate time points. The drastic increase of *I*-score as well as the largest value both appeared in the red curve, indicating the maximum likelihood of 8 h being the critical point. It is also clear that there was a drastic increase of *I*-score around 4 h, which reached a peak at 8 h for the red curve, signalling the imminent critical transition around 4–8 h, and thus indicating the pre-disease stage just before the deterioration into serious lung pulmonary injury. As mentioned in the Methods section, the *I*-score for the real dataset was an average of local scores based on local differential networks, each of which contain a centre node (a differentially-expressed gene) and all of its first-order neighbours based on STRING network structure. To illustrate the relevance of these local differential networks, the local *I*-scores at three time points (1, 8, and 48 h) from case and control samples respectively are presented in Fig. 3b, where each local network is labelled by its centre gene. Obviously, the local *I*-score from case samples demonstrate the sensitivity and significance at the identified transition point (8 h). Furthermore, Fig. 3c illustrates the dynamical changes of the whole mouse molecular network from 0.5 to 48 h, where a drastic change in terms of expression variations and network structure of a group of genes located in the lower-right corner can be observed around 8 h. In Fig. 3c, the temporal alteration of the network is exhibited through differential variations and correlations, based on which the differential network construction are constructed. The detailed calculating procedure are presented in Additional file 1: C.

Clearly, from Fig. 3a–c, when deterioration is impending, the abrupt change in *I*-score implies a warning signal at the 4–8 h period. Conversely, the *I*-score is sensitive in the pre-disease state, since there is no obvious indicative sign or dynamics either in normal state or disease state. Reminding that the *I*-score signal is yielded from the collective behavioral dynamics of members in the differential network, the pre-disease state is hardly detected by any single biomolecules. Therefore, it may make the identification and management of high-risk cases more effective by applying the *I*-score scheme to the detection of pre-disease stage.

**Fig. 4:**
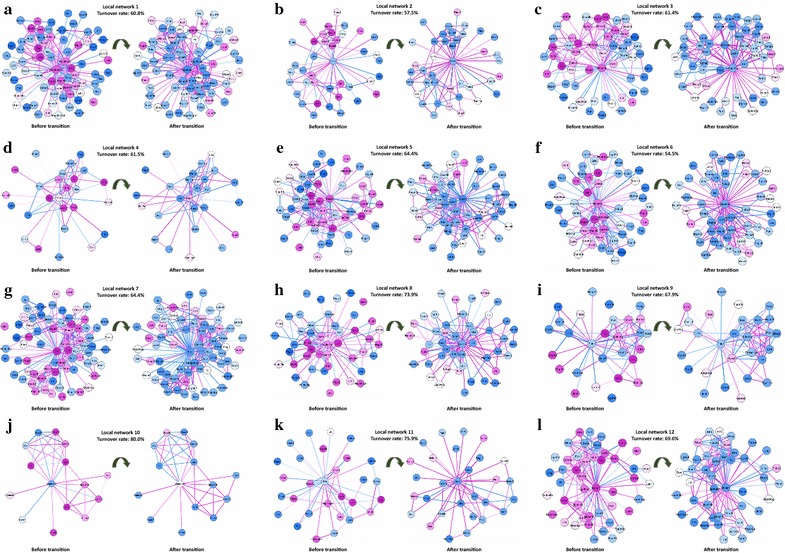
Demonstration of 12 pairs of significant local differential networks. To demonstrate the effectiveness of the *I*-score scheme, 12 pairs of the most significant differential local networks are presented, i.e., local networks with dynamically significant change in *I*-scores around the identified critical time point (8 h). For each pair, the left network is the differential network in the normal state (4 h), while the right one is in the disease state (12 h). In terms of these networks, 55–80% nodes had turnover (from low expression to high expression with significance value *P* < 0.05, or vice versa) and 33–60% edges had turnover (from negative correlation value to positive, or vice versa) when the system progressed from the normal state to the disease state. The ratios of the turnover neighbours and edges overwhelm those of the background 28.6% (turnover neighbours) and 18.1% (turnover edges), i.e., the ratio of the turnover nodes/edges in the whole STRING molecular network. Among these significant local networks, some well-known genes that were involved in apoptosis or related to the inflammatory response were included: JUN (local network 8), NOTCH2 (local network 12), MYC (local network 1), IL1B (local network 7), and PTGS2 (local network 5). To analyse and illustrate the dynamical difference before and after the critical transition, graph-related information is shown in Table 1

To further validate the identified critical point and elucidate the local differential networks with apparent turnover features, we presented 12 pairs of local networks with dynamically significant changes in *I*-scores around the identified critical time point at 8 h (Fig. 4). These local networks were mapped to an integrated STRING network. Each turnover gene (node) or turnover regulation (edge) expressed lowly (or highly) before the tipping point and highly (or lowly) afterward. The mass turnover features of these local networks demonstrate the biological significance of the identified critical point. The graph-related information is shown in Table 1, from which it can be seen that the structures of these networks changes drastically between normal and disease states. The ratios of the turnover neighbours and edges overwhelm those of the background 28.6% (turnover neighbours) and 18.1% (turnover edges), i.e., the ratio of the turnover nodes/edges in the whole STRING molecular network.

**Table 1:** Graph-related information of twelve networks with most significant *I*-scores

Network	Centre gene	Number of nodes	Turnover edges	Ratio of turnover edges	Turnover nodes	Ratio of turnover nodes	Significance
1	Hsp90ab1	74	26	0.36	45	0.61	2.0E−6
2	Slc2a1	40	13	0.33	23	0.58	7.0E−6
3	Hspa1b	57	25	0.45	35	0.61	2.0E−5
4	Thbs1	26	15	0.60	16	0.62	3.8E−4
5	Ptgs2	55	26	0.48	35	0.64	1.9E−3
6	Hspa1a	55	32	0.59	30	0.55	7.3E−4
7	Fos	73	29	0.40	47	0.64	5.1E−3
8	Cdkn1a	46	15	0.33	34	0.74	3.5E−5
9	Plaur	28	11	0.41	19	0.68	1.4E−5
10	Gadd45g	15	5	0.36	12	0.80	1.6E−5
11	Gclc	29	11	0.39	22	0.76	0
12	Dusp1	46	15	0.33	32	0.70	1.9E−3

Briefly, these analysis implied that during the first 8 h after inhaling phosgene, the main pathological process of the case group resulted in an enhancement of bronchoalveolar lavage fluid protein levels, triggered pulmonary edema, and eventually increased death rates [30]. The severe phosgene-induced acute lung injury was around 8 h and lasted until 12 h after exposure. As the phosgene exposure continued, 50–60% mortality occurred after 12 h and 60–70% mortality was observed after 24 h [30]. Indicative signals of severe injury through *I*-score scheme are shown in Fig. 3a, illustrating that the onset of pre-disease state is around 8 h, and the system steps into the disease state (pulmonary edema) after 12 h. Our prediction based on the *I*-score was in agreement with the observations of the experiment. To better show the significance of the results, the validation *I*-score was calculated based on a leave-one-out bootstrap procedure in Additional file 1: Figure S3. Based on each set of re-sampled data, the similar increasing trend of *I*-score curve around 4–8 h also demonstrated the consistence of the approach.

The second application of *I*-score was for a time-course genomic data of acute corneal trauma (GSE1393), which was generated from a mouse model with acute corneal trauma induced by a chemical burn to the cornea with silver nitrate, to study ophthalmic organ damage, overstimulation, and the related gene regulation. In the experiment, the RNA of the lacrimal gland was extracted from female BALB/c mice bilaterally cauterized with silver nitrate, thus generated the case data at sampling point, i.e., 0.5, 1, 3, 8, 24, 72, 120, and 360 h after the corneal burn.

**Fig. 5:**
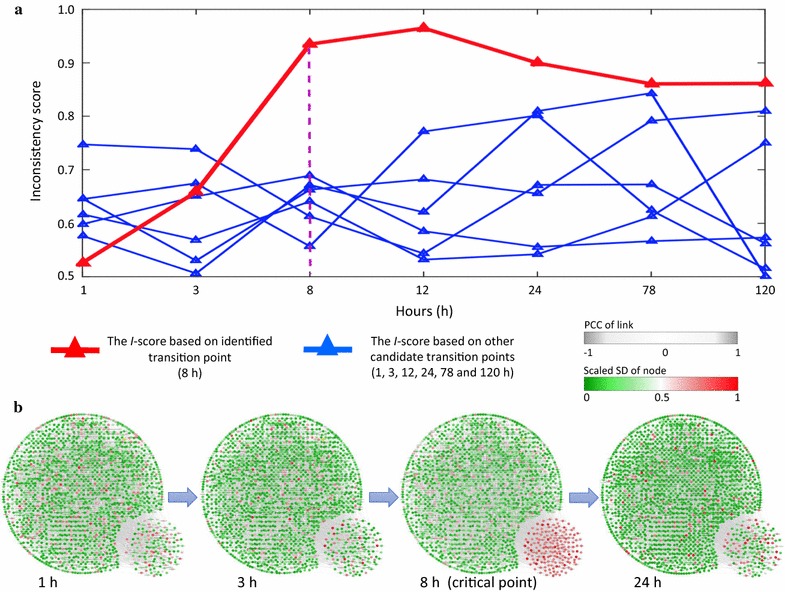
Application of $\textit{I}$-score on the dataset of acute corneal trauma. **a** The *I*-score based on microarray of acute corneal trauma from each candidate transition time point. The red curve represents the *I*-score calculated from the testing differential network obtained 8 h after acute corneal trauma, while the six blue curves are those from other time points. The abrupt increase of *I*-score appeared around the 1–8 h period, which is in coincidence with the experimental observation, i.e., the heat shock genes were upregulated beginning at 8 h, indicating the start of a stress response. **b** A group of 171 genes with top 10% most significant *I*-scores’ change are located in the lower-right corner in each network. These selected genes showed wild fluctuation in their expressions around the 8 h time point. Thus, the critical transition point was around 8 h, where the network structure exhibits the most significant change, just before the critical transition triggered by acute corneal trauma

We then exhibited the application of *I*-score for acute corneal trauma. In Fig. 5a, each *I*-score curve is calculated from an HMM that obtained based on a set of differential networks yielded before a candidate critical time point. The red curve in Fig. 5a shows a drastic increase of *I*-score around 1–3 h and reach the peak at the 8-h sampling point, which implies that 8 h is a tipping point with the highest probability. To demonstrate the system progression at the network level, four consequent whole gene regulatory network were illustrated on the basis of the case data of acute corneal trauma in Fig. 5b, from which the network structure also showed a significant change at 8 h. In the original experiment, the heat shock genes were upregulated beginning at the 8 h time point, indicating the start of a stress response [31]. The changes in gene expression attained two peaks at 8 and 72 h, and then declined to low levels of activity at later time points. In line with the actual observation, our analysis successfully identified the critical state before the emergence of strong stress response.

The third application of *I*-score on the heregulin-induced breast cancer (GSE13009) is demonstrated in Additional file 1: Figure S4 and related contents placed in Additional file 1: C.

### Functional analysis

As one of the most important and common chemical industry compounds, the phosgene gas and its inhalation damage receives extensive attention [33]. Some pathogenic and genomic mechanisms of the pulmonary injury activated by phosgene exposure have been studied [30]. From our analysis, a significant change in the average *I*-score of the local differential networks occurred from 4 to 8 h. Analysing the genes from local differential networks with dynamically significant change in *I*-score (Fig. 4), pathway enrichment and gene ontology functional analysis suggested that the genes in these local networks are highly related to the mechanism of disease progression [30, 34]. Additionally, some well-known genes involved in apoptosis or related to the inflammatory response were included, such as JUN (local network 8), NOTCH2 (local network 12), MYC (local network 1), IL1B (local network 7), and PTGS2 (local network 5) (Fig. 4), which may reduce the number of injured cells.

Moreover, some genes in the identified local differential networks were consistent with the dysfunction in glutathione metabolism (such as ASNS and GCLC) and the inflammatory immune response associated with chemokine signalling pathway (such as IL1B), which may relate to the abnormality of antioxidant reactions (such as PTGS2) caused by continually inhaling of phosgene. Thus, resulted from oxidant reaction of carbonyl chloride and the decrease of antioxidant enzyme concentrations, some protein-modified signalling pathways, including the Wnt signalling pathway and the mitogen-activated protein kinase (MAPK) signalling pathway, were disordered and thus may affect pro-inflammatory cytokines and other inflammatory mediators (such as NFKB1) [35].

The enrichment analysis also suggested that some signalling pathways may also be involved or affected in the phosgene-induced pathogenic processes, such as TGF-beta signalling pathway and hormone-mediated signalling pathway, which associated with the cell repair and reproduction.

Besides, some biological processes were also suggested to be highly relevant to lung injury through GO functional analysis, i.e., the differentially high/low expression of genes in the identified local differential networks associated with abnormal alterations in primary metabolic processes, which indicates the denaturation of lipids, proteins, and nucleic acids that may have been oxidized by phosgene [30, 34].

The functional analyses for acute corneal trauma and heregulin-induced breast cancer are shown in Additional file 1: C.

## Discussion

Signalling the abrupt critical transition of complex diseases is crucial to patients all over the world. The irreversible deterioration may thus be prevented or at least delayed by timely interference. However, it is challenging to distinguish the pre-disease samples from normal samples and detect the imminent qualitative transition, not only because there is usually little obvious state change before reaching the tipping point, but the disease initiation dynamics before sudden deterioration is generally too complex to be fully expressed mathematically in high dimensional spaces. To overcome these difficulties, new methods are expected in both extracting discriminative dynamical features from biomolecules as relevant as possible for describing the progression of a biological system, and developing an applicable unsupervised learning-testing algorithm to detect the signal of the upcoming catastrophic transition into a disease state.

In this work, different from conventional approaches based on the differential expression of observed biomolecules, we presented a computational method with an inconsistency score from constructing a temporal sequence of differential networks for a better understanding of the dynamical progression when a biological system is near the critical transition point. The rewiring of differential networks effectively extracts discriminatively interpretable features, and systematically demonstrates the dynamical change of a biological system. In view of that, the temporal sequence of differential networks may be ideal study object for understanding the functional alteration during the progression of a complex disease. Furthermore, we proposed a computational method based on machine learning to signal the upcoming critical transition. The applications on both numerical experiment (Fig. 2) and microarray data from three pathological experiments (Figs. 3, 4, 5 and Additional file 1: Figure S4) validated the effectiveness of the method. Unlike the diagnosis of the disease state in which catastrophic transition has already occurred, our method enables the identification of an intermediate or transition state that generally has no clear abnormalities but with future trending of deterioration. The DNB concept theoretically provided several statistical properties when the system approaches a tipping point, based on which the applicable inconsistency score was developed to quantitatively identify the pre-disease stage. For example, the initial lung damage of mice is not generally detectable until the emergence of pulmonary edema due to continually phosgene inhalation, an indicative early-warning signal yielded from the inconsistency score demonstrated the existence of the pre-disease state around 4–8 h after exposed to phosgene gas, prior to the serious deterioration. This specific state is thus described as the prelude to the critical transition into the disease state.

From these applications it can be seen that, the differential network based algorithm is an effective aggregate method capable of exploiting the high-dimensional information from the longitudinal measurements of molecular factors involved in a disease, and better record the dynamical change when the data is perturbed by noises (Additional file 1: Figure S2). Further, at differential-network level, the *I*-score scheme, which is model-free and works even we do not know which variable or subnetwork is important, provided a computational way of prying into the underlying mechanism of the dynamical progression of disease in the vicinity of a tipping point and thus helping to achieve timely intervention. This method and algorithm is therefore much more robust in detecting the tipping point during the biological processes than our previous methods [15, 21, 25], and thus develop the DNB strategy.

There are limitations to this work. First, the validity of the identified critical state and the underlying biological mechanism require further support from animal experiments or clinical studies. Second, the accuracy of this method is dependent on the amount of samples in each time point/stage, since the construct of the differential network relies on statistical indices, which might be inaccurate when there are only small samples. Although this work is merely a step toward detecting the indicative signals of a critical transition during disease progression and the algorithm is expected to be improved in both sensitivity and accuracy, it presents a new way for identifying an imminent deterioration in the differential-network level, and may bring further downstream implications.

## Conclusion

In summary, we proposed a computational method to detect the early-warning signal of the impending critical transition during biological process of complex diseases. The scheme of inconsistency score is expected to be of wide range of applicable potential in future biological and clinical studies, since it possesses advantages such as model-free and robustness, due to its theoretical background closely related to the dynamical change of the biomolecular network. However, how to apply this method under the circumstance of small samples remains to be studied.

## Additional file

**Additional file 1.** Detecting pre-disease states by temporal differential network.

## Abbreviations

HMM: hidden Markov model
*I*-score: inconsistency score
DNB: dynamical network biomarker
SD: standard deviation
PCC: Pearson’s correlation coefficients

## References

[1] Liu R, Wang X, Aihara K, Chen L (2014). Early diagnosis of complex diseases by molecular biomarkers, network biomarkers, and dynamical network biomarkers. Med Res Rev.

[2] Scheffer M, Bascompte J, Brock WA, Brovkin V, Carpenter SR, Dakos V (2009). Early-warning signals for critical transitions. Nature.

[3] He D, Liu ZP, Honda M, Kaneko S, Chen L (2012). Coexpression network analysis in chronic hepatitis B and C hepatic lesions reveals distinct patterns of disease progression to hepatocellular carcinoma. J Mol Cell Biol.

[4] Tan Z, Liu R, Zheng L, Hao S, Fu C, Li Z (2015). Cerebrospinal fluid protein dynamic driver network: at the crossroads of brain tumorigenesis. Methods.

[5] Achiron A, Grotto I, Balicer R, Magalashvili D, Feldman A, Gurevich M (2010). Microarray analysis identifies altered regulation of nuclear receptor family members in the pre-disease state of multiple sclerosis. Neurobiol Dis.

[6] Chen L, Liu R, Liu ZP, Li M, Aihara K. Detecting early-warning signals for sudden deterioration of complex diseases by dynamical network biomarkers. Sci Rep. 2012;2:342. 10.1038/srep00342 PMC3314989 22461973

[7] Liu R, Aihara K, Chen L (2013). Dynamical network biomarkers for identifying critical transitions and their driving networks of biologic processes. Quant Biol.

[8] Litt B, Esteller R, Echauz J, Alessandro MD, Shor R, Henry T (2001). Epileptic seizures may begin hours in advance of clinical onset: a report of five patients. Neuron.

[9] Venegas JG, Winkler T, Musch G, Melo MF, Layfield D, Tgavalekos N (2005). Self-organized patchiness in asthma as a prelude to catastrophic shifts. Nature.

[10] McSharry PE, Smith LA, Tarassenko L (2003). Prediction of epileptic seizures: are nonlinear methods relevant?. Nat Med.

[11] Roberto PB, Eliseo G, Josef C (2003). Transition models for change-point estimation in logistic regression. Statist Med.

[12] Paek SH, Chung HT, Jeong SS, Park C, Kim C, Kim JE (2005). Hearing preservation after gamma knife stereotactic radiosurgery of vestibular schwannoma. Cancer.

[13] Liu JK, Rovit RL, Couldwell WT (2001). Pituitary apoplexy. Semin Neurosurg.

[14] Barabasi A-L, Gulbahce N, Loscalzo J (2011). Network medicine: a network-based approach to human disease. Nat Rev Genet.

[15] Liu R, Li M, Liu ZP, Aihara K, Chen L (2012). Identifying critical transitions and their leading biomolecular networks in complex diseases. Sci Rep.

[16] Liu R, Yu X, Liu X, Xu D, Aihara K, Chen L (2014). Identifying critical transitions of complex diseases based on a single sample. Bioinformatics.

[17] Li M, Zeng T, Liu R, Chen L (2014). Detecting tissue-specific early warning signals for complex diseases based on dynamical network biomarkers: study of type 2 diabetes by cross-tissue analysis. Brief Bioinform.

[18] Chen P, Liu R, Chen L, Aihara K (2015). Identifying critical differentiation state of MCF-7 cells for breast cancer by dynamical network biomarkers. Front Genet.

[19] Liu R, Chen P, Aihara K, Chen L. Identifying early-warning signals of critical transitions with strong noise by dynamical network markers. Sci Rep. 2015;5:17501. 10.1038/srep17501 PMC4673532 26647650

[20] Liu X, Liu R, Zhao XM, Chen L (2013). Detecting early-warning signals of type 1 diabetes and its leading biomolecular networks by dynamical network biomarkers. BMC Med Genom.

[21] Chen P, Liu R, Li Y, Chen L (2016). Detecting critical state before phase transition of complex biological systems by hidden Markov model. Bioinformatics.

[22] Mojtahedi M, Skupin A, Zhou J, Castano IG, Leong-Quong RYY (2016). Cell fate decision as high-dimensional critical state transition. PLoS Biol.

[23] Richard A, Boullu L, Herbach U, Bonnafoux A, Morin V (2016). Single-cell-based analysis highlights a surge in cell-to-cell molecular variability preceding irreversible commitment in a differentiation process. PLoS Biol.

[24] Lesterhuis WJ, Bosco A, Millward MJ (2017). Dynamic versus static biomarkers in cancer immune checkpoint blockade: unravelling complexity. Nat Rev Drug Discov.

[25] Chen P, Li Y (2016). The decrease of consistence probability: at the crossroad of catastrophic transition of a biological system. BMC Syst Biol.

[26] Gilmore R (1993). Catastrophe theory for scientists and engineers.

[27] Schadt EE (2009). Molecular networks as sensors and drivers of common human diseases. Nature.

[28] Liu X, Liu ZP, Zhao XM, Chen L (2012). Identifying disease genes and module biomarkers by differential interactions. J Am Med Inform Assoc.

[29] Chen L, Wang RS, Zhang XS (2009). Biomolecular networks: methods and applications in systems biology.

[30] Sciuto AM, Phillips CS, Orzolek LD, Hege AI, Moran TS, Dillman JF (2005). Genomic analysis of murine pulmonary tissue following carbonyl chloride inhalation. Chem Res Toxicol.

[31] Fang Y, Choi D, Searles RP, Mathers WD (2005). A time course microarray study of gene expression in the mouse lacrimal gland after acute corneal trauma. Invest Ophthalmol Vis Sci.

[32] Saeki Y (2009). Ligand-specific sequential regulation of transcription factors for differentiation of MCF-7 cells. BMC Genom.

[33] Schneider W, Diller W (2000). Phosgene, in Ullmann’s Encyclopedia of Industrial Chemistry.

[34] Wang P, Ye XL, Liu R, Chen HL, Liang X, Li WL (2013). Mechanism of acute lung injury due to phosgene exposition and its protection by cafeic acid phenethyl ester in the rat. Exp Toxicol Pathol.

[35] Herlaar E, Brown Z (1999). p38 MAPK signalling cascades in inflammatory disease. Mol Med Today.

