# Supplementary material for: Detecting the tipping points in a three-state model of complex diseases by temporal differential networks
Source: J Transl Med. 2017 Oct 26;15:217. doi: 10.1186/s12967-017-1320-7 (PMC5658963; doi:10.1186/s12967-017-1320-7)
Supplement: Supplementary file 1 — Additional file 1. Detecting pre-disease states by temporal differential network. [file 12967_2017_1320_MOESM1_ESM.pdf]

# Supplementary Information: Detecting Pre-disease States by Temporal Differential Network

Rui Liu, Pei Chen, Yongjun Li, Xiaoping Liu, Luonan Chen

## Contents

**A Theoretical background** **S2**

A.1 Theoretical basis near critical transition point . . . . . . . . . . . . . . . . . . . S2

A.2 Three states during a critical transition . . . . . . . . . . . . . . . . . . . . . . . S8

A.3 Identifying the switching point of stationary Markov process based on inconsistency score . . . . . . . . . . . . . . . . . . . . . . . . . . . . . . . S8

A.3.1 Algorithm . . . . . . . . . . . . . . . . . . . . . . . . . . . . . . . S9

A.3.2 Deriving the HMM based on an unsupervised learning procedure . . . S10

A.3.3 Calculating the inconsistency score at a testing time point $t = T$ . . . . S16

**B Numerical validation of $I$-score** **S18**

**C Application to three real datasets** **S22**

C.1 Dataset 1. Genomic data of the lung injury with carbonyl chloride inhalation exposure (i.e., acute lung injury) . . . . . . . . . . . . . . . . . . . . . . . . . S23

C.2 Dataset 2: Genomic data on acute corneal trauma . . . . . . . . . . . . . . . . S31

C.3 Dataset 3: Genomic data of MCF-7 human breast cancer caused by heregulin (HRG) . . . . . . . . . . . . . . . . . . . . . . . . . . . . . . . . . . . S32

# A Theoretical background

## A.1 Theoretical basis near critical transition point

For a complex dynamical system with multiple variables or network, assume that we measure the variables at different time points. In this section, we theoretically introduce several generic properties of such a dynamical network when the system approaches a critical transition point. Specifically, we derive the conditions to obtain dynamical network biomaker (DNB) in a biological system, which can characterize the generic properties and predict the critical transition, based on bifurcation theory and center manifold theory (*6, 7*).

We consider the following discrete-time dynamical system that represents the dynamical evolution of a network:

$$Z(t+1) = f(Z(t); P), \tag{S1}$$

where $Z(t) = (z_1(t), ..., z_n(t))$ is an $n$-dimensional state vector or variables at time instant $t$, while $P = (p_1, ..., p_s)$ is a parameter vector or driving factors that represent slowly changing factors, e.g., genetic factors (SNP, CNV, etc.) and epigenetic factors (methylation, acetylation, etc.). $f : \mathbf{R}^n \times \mathbf{R}^s \to \mathbf{R}^n$ are generally nonlinear functions.

Furthermore, we assume that the following conditions hold for Eq.(S1).

1. $\bar{Z}$ is a fixed point in system (S1) such that $\bar{Z} = f(\bar{Z}; P)$.

2. There is a value $P_c$ such that one or a pair of the eigenvalues of the Jacobian matrix $\left.\frac{\partial f(Z;P_c)}{\partial Z}\right|_{Z=\bar{Z}}$ is equal to 1 in the modulus.

3. When $P \neq P_c$, the eigenvalues of (S1) are not always equal to 1 in the modulus.

These three assumptions with other transverse conditions imply that the system undergoes a phase change at $\bar{Z}$ or a codimension-one bifurcation when $P$ reaches the threshold $P_c$ (*1*).

From a mathematical perspective, the bifurcation is generic, *i.e.* almost all of the bifurcations in a general system satisfy these conditions. It is notable that most of the systems described by differential equations can be generally discretized and transformed into Eq.(S1), e.g., using methods such as the Euler scheme and the Poincaré section. Thus, we focus on difference equations (S1) during our theoretical analysis in this section.

It is known that the dynamics of a nonlinear system is highly complex far before or after a sudden transition; therefore, the state equations of systems are generally constructed in a very high-dimensional space using a large number of variables and parameters (*1–4, 12, 14*). However, if a system driven by known or unknown parameters approaches a critical point, which is a very special phase during its dynamical evolution, it is theoretically guaranteed that the system will eventually be constrained to one- or two-dimensional space (i.e., the center manifold), which can be expressed in a simple form around a codimension-one bifurcation point (*5,6,12,14*). This is generally guaranteed by the bifurcation theory and the center manifold theory (*5–8*). Thus, we can detect the signal of any dynamical system only during this special phase and not during other periods (i.e., neither the before-transition state nor the after-transition state), which is part of the theoretical foundation of this study (*12, 14*).

For system (S1) near $\bar{Z}$ and before $P$ reaches $P_c$, we assume that the system is at a stable fixed point $\bar{Z}$, so all of the eigenvalues are within $(0, 1)$ in the modulus. The parameter value $P_c$ at which the state shift of the system occurs, is known as a bifurcation parameter value or a critical transition value.

This theoretical result was derived based on consideration of the linearized system or equations for Eq.(S1) and the small noise perturbations near $\bar{Z}$. Specifically, by introducing the new variables $Y(t) = (y_1(t), ..., y_n(t))$ and a transformation matrix $S$, *i.e.*

$$Y(t) = S^{-1}(Z(t) - \bar{Z}),$$

or

$$z_i(k) = s_{i1}y_1(k) + \cdots + s_{in}y_n(k) + \bar{z}_i, \quad k, i = 1, 2, ..., n, \tag{S2}$$

we have

$$Y(t+1) = \Lambda Y(t) + \zeta(t). \tag{S3}$$

where $\Lambda(P)$ is the diagonalized matrix of $\frac{\partial f(Z;P)}{\partial Z}\Big|_{Z=\bar{Z}}$. $\zeta(t) = (\zeta_1(t), ..., \zeta_n(t))$ are small Gaussian noises with zero means. Without any loss of generality, the diagonalized matrix $\Lambda(P) = diag(\lambda_1(P), ..., \lambda_n(P))$ for each $|\lambda_i|$ is between 0 and 1. Denote the dominant eigenvalue as the largest eigenvalue or eigenvalues (the case of multiple roots) in modulus. Here, the largest eigenvalue (in the sense of modulus) characterizes the system's rate of change around a fixed point and is known as the dominant eigenvalue. The before-transition state corresponds to a period when the dominant eigenvalue is smaller than 1 in modulus, whereas the pre-transition stage corresponds to the period with the dominant eigenvalue approaching to 1 in modulus. Actually, in view of the dominant eigenvalue, there are two typical cases during the diagonalization process (*12*), i.e., the dominant eigenvalue is real (including multiple real dominant eigenvalues), and the dominant eigenvalues are a pair of complex conjugate values.

When the modulus of the largest eigenvalue or eigenvalue pairs approaches 1, there are three generic codimension-one bifurcations of the system, corresponding to these cases, that is the saddle-node bifurcation, period-doubling bifurcation and Neimark-Sacker bifurcation. Specifically, when the dominant eigenvalue is real, the critical point is the saddle-node bifurcation (transcritical and pitchfork bifurcation) if the dominant eigenvalue approaches 1, while the critical point is the period-doubling (or flip) bifurcation if the dominant eigenvalue approaches -1. When the dominant eigenvalues are a pair of complex conjugate eigenvalues (including several pairs with the same modulus), for such a case, the critical point is the Neimark-Sacker bifurcation point (*1*).

According to our previous works (*12, 14*), we have the following results.

1. When the dominant eigenvalues are real, then there is only one dominant group related to the first variable $y_1$ in Eq.(S3), which is with standard deviation

$$\mathrm{SD}(z_i) = \sqrt{s_{i1}^2 \frac{\kappa_{11}}{1-\lambda_1^2} + \sum_{k=2}^{n} s_{ik}^2 \frac{\kappa_{kk}}{1-\lambda_k^2} + \sum_{k,m=1,k\neq m}^{n} s_{ik}s_{im}\frac{\kappa_{km}}{1-\lambda_k\lambda_m}},$$

and Pearson's coefficient correlation

$$\mathrm{PCC}(z_i, z_j) =$$

$$= \frac{s_{i1}s_{j1}\frac{\kappa_{11}}{1-\lambda_1^2} + \sum_{k=2}^{n} s_{ik}s_{jk}\frac{\kappa_{kk}}{1-\lambda_k^2} + \sum_{k,m=1,k\neq m}^{n} s_{ik}s_{jm}\frac{\kappa_{km}}{1-\lambda_k\lambda_m}}{\sqrt{\left(\frac{s_{i1}^2\kappa_{11}}{1-\lambda_1^2} + \sum_{k=2}^{n}\frac{s_{ik}^2\kappa_{kk}}{1-\lambda_k^2} + \sum_{k,m=1,k\neq m}^{n}\frac{s_{ik}s_{im}\kappa_{km}}{1-\lambda_k\lambda_m}\right)\left(\frac{s_{j1}^2\kappa_{11}}{1-\lambda_1^2} + \sum_{k=2}^{n}\frac{s_{jk}^2\kappa_{kk}}{1-\lambda_k^2} + \sum_{k,m=1,k\neq m}^{n}\frac{s_{jk}s_{jm}\kappa_{km}}{1-\lambda_k\lambda_m}\right)}},$$

where $\lambda_1$ represents the dominant eigenvalue while $\lambda_k$ is other eigenvalue with $|\lambda_k| \leq |\lambda_1|$, the constant $s_{jk}$ is the coefficient in Eq.(S2), and $\kappa_{ij}$ is the covariances between Gaussian noises $\zeta_i$ and $\zeta_j$ in Eq.(S2).

2. When the dominant eigenvalues are a pair of complex conjugate eigenvalues, then there are two dominant groups respectively related to $y_1$ and $y_2$ in Eq.(S3). The standard deviation for the variables $z_i$ is

$$\mathrm{SD}(z_i) = \sqrt{\frac{2b^2(s_{i1}^2 + s_{i2}^2)(\kappa_{11}+\kappa_{22})}{(1-a^2-b^2)((a-1)^2+b^2)((a+1)^2+b^2)} + K_i}$$

where $K_i$ are bounded values, and Pearson's coefficient correlation

$$\mathrm{PCC}(z_i, z_j) =$$

$$= \frac{\left(\frac{2b^2(s_{i1}s_{j1}+s_{i2}s_{j2})(\kappa_{11}+\kappa_{22})}{(1-a^2-b^2)((a-1)^2+b^2)((a+1)^2+b^2)}\right) + C_{ij}}{\sqrt{\left(\frac{2b^2(s_{i1}^2+s_{i2}^2)(\kappa_{11}+\kappa_{22})}{(1-a^2-b^2)((a-1)^2+b^2)((a+1)^2+b^2)} + K_i\right)\left(\frac{2b^2(s_{j1}^2+s_{j2}^2)(\kappa_{11}+\kappa_{22})}{(1-a^2-b^2)((a-1)^2+b^2)((a+1)^2+b^2)} + K_j\right)}}.$$

where $C_{ij}$, $K_i$ and $K_j$ are bounded values.

**Theorem 1** *We consider a stochastically perturbed linearized system for Eq.(S1). When $P$ approaches the saddle-node or period-doubling bifurcation point, there is a dominant group, and the following results hold.*

- *If both $z_i$ and $z_j$ are in the dominant group, then*

$$|\mathrm{PCC}(z_i, z_j)| \to 1,$$

  *while $\mathrm{SD}(z_i) \to \infty$ and $\mathrm{SD}(z_j) \to \infty$;*

- *if $z_i$ is in the dominant group but $z_j$ is not, then*

$$\mathrm{PCC}(z_i, z_j) \to 0,$$

  *while $\mathrm{SD}(z_i) \to \infty$, and $\mathrm{SD}(z_j)$ approaches a bounded value;*

- *if neither $z_i$ nor $z_j$ is in the dominant group, then $\mathrm{PCC}(z_i, z_j)$ approaches a constant, while both $\mathrm{SD}(z_i)$ and $\mathrm{SD}(z_j)$ approach bounded values,*

*where PCC is the Pearson's correlation coefficient and SD is the standard deviation.*

This dominant group of variables or elements is DNB. This theorem is the part of the theoretical basis of detecting the pre-transition state for multi-variable systems with small noise. The three conditions in the theorem are actually the criteria to detect the DNB (*12*). For a nonlinear system Eq.(S1) approaching the bifurcation point (the saddle-node or period-doubling bifurcation point) or tipping point, we can observe directly obtain the properties of DNB based on Theorem 1 as the following remark.

**Remark 1** *For nonlinear case Eq.(S1) near a bifurcation point (the saddle-node or period-doubling bifurcation point), the dynamical behavior has the same tendency as that of the linearized case, that is, when the system is approaching to the bifurcation point, both indices*

$\mathrm{SD}$ *and* $|\mathrm{PCC}|$ *in the DNB increase sharply, while* $|\mathrm{PCC}|$ *between DNB and other non-DNB molecules decreases rapidly, i.e.,*

1. *If both* $z_i$ *and* $z_j$ *are in the DNB, then* $\mathrm{PCC}(z_i, z_j)$ *increases, while* $\mathrm{SD}(z_i)$ *and* $\mathrm{SD}(z_j)$ *drastically increase;*

2. *if* $z_i$ *is in the DNB but* $z_j$ *is not, then* $\mathrm{PCC}(z_i, z_j)$ *decreases, while* $\mathrm{SD}(z_i)$ *drastically increases, and there is no significant change for* $\mathrm{SD}(z_j)$*;*

3. *if neither* $z_i$ *nor* $z_j$ *is in the DNB, then there are no significant changes on* $\mathrm{PCC}(z_i, z_j)$*,* $\mathrm{SD}(z_i)$ *and* $\mathrm{SD}(z_j)$*.*

**Theorem 2** *We consider a stochastically perturbed linearized system for Eq.(S1). When* $P$ *approaches the Neimark-Sacker bifurcation point, the following results hold.*

- *If both* $z_i$ *and* $z_j$ *are in the same dominant group 1 or 2, then*

$$|\mathrm{PCC}(z_i, z_j)| \rightarrow 1,$$

  *while* $\mathrm{SD}(z_i) \rightarrow \infty$ *and* $\mathrm{SD}(z_j) \rightarrow \infty$;

- *if* $z_i$ *is in dominant group 1 and* $z_j$ *in dominant group 2, then*

$$\mathrm{PCC}(z_i, z_j) \rightarrow 0,$$

  *while* $\mathrm{SD}(z_i) \rightarrow \infty$*, and* $\mathrm{SD}(z_j) \rightarrow \infty$*;*

- *if* $z_i$ *is in a dominant group (including dominant 1 or 2, common-dominant group) and* $z_j$ *is in the non-dominant group, then*

$$\mathrm{PCC}(z_i, z_j) \rightarrow 0,$$

  *while* $\mathrm{SD}(z_i) \rightarrow \infty$ *and* $\mathrm{SD}(z_j)$ *approaches a bounded value;*

- *if $z_i$ is in the common dominant group, and $z_j$ is in dominant group $k$ $(k = 1, 2)$ or the common dominant group, then $|\mathrm{PCC}(z_i, z_j)|$ approaches a constant less than 1, while $\mathrm{SD}(z_i) \to \infty$, and $\mathrm{SD}(z_j) \to \infty$;*

- *if neither $z_i$ nor $z_j$ is in the dominant group, then $|\mathrm{PCC}(z_i, z_j)|$ approaches a constant less than 1, while both $\mathrm{SD}(z_i)$ and $\mathrm{SD}(z_j)$ approach bounded values;*

*where PCC is the Pearson's correlation coefficient and SD is the standard deviation.*

**Remark 2** *When both $z_i$ and $z_j$ are in the common dominant group, if their relations to $y_1$ are much more stronger than that to $y_2$, then generally $z_i$ and $z_j$ are considered in dominant group 1. Contrarily, if the relations of $z_i$ and $z_j$ to $y_2$ are much more stronger than that to $y_1$, then generally $z_i$ and $z_j$ are considered in dominant group 2.*

## A.2 Three states during a critical transition

During a state transition, the dynamics of the system can be divided as three stages. Before-transition state corresponds to a stable equilibrium. In bio-medical systems, it generally corresponds to a "normal state" or a stable period that the disease is under control. Pre-transition state is the limit of Before-transition state. In bio-medical systems, it represents a "pre-disease state" just before the critical transition to the disease state. After-transition state corresponds to another stable equilibrium. In bio-medical systems, it represents a badly ill stage or "disease state", and is usually difficult to return to the before-transition state even by big perturbations.

## A.3 Identifying the switching point of stationary Markov process based on inconsistency score

In our assumption, the stage before the transition point is quite different from that after the transition point. Therefore, we regard that the progression of a biological system in the before-transition stage is a stationary Markov process, since during this stage the system is stable and

insensitive to parameter changes (stationary feature). However, the progression of a biological system in the pre-transition stage is modelled as a time-varying Markov process, due to the strong fluctuated dynamics during this stage, i.e., the system is unstable and so sensitive to the parameter changes that even a small change in the parameters may suffice to drive the system into collapse (time-varying feature) (*24*). Therefore, to detect the onset of the pre-transition stage is equivalent to identify the changing period or the switching point from a stationary Markov process to a time-varying Markov process.

On the basis of above settings and hypothesis, we propose an inconsistency score ($I$-score) to measure the probability of a candidate time point as the changing or switching point from the stationary Markov process to the time-varying Markov process. Specifically, we test each candidate time point based on an HMM derived from an unsupervised learning procedure.

### A.3.1 Algorithm

There are two steps to detect the switching period in our algorithm, i.e.,

- Training step: In the training step, obtain the HMM $\Theta_{T-1} = (A, B, \pi)$ based on the preceding $T-2$ differential network series $\{DN_2, DN_3, ..., DN_{T-1}\}$, which are constructed based on observed samples from preceding $T-1$ sampling time points. Here $A = (a_{ij}(T-1))_{2\times 2}$ with

$$a_{ij}(T-1) = P(s_{T-1} = W_i \,|\, s_{T-2} = W_j), \quad i, j \in \{0, 1\}, \tag{S4}$$

  $B = (b_{jk}(T-1))_{2\times(n+1)}$ with $b_{jk}(T-1)$ representing the probability of the $k$th possible observation under the assumption that the system state is $W_j, \ \ j \in \{0, 1\}$ at time $T-1$ i.e.,

$$b_{jk}(t-1) = P(\#1(T-1) = k \,|\, s_{T-1} = W_j), \quad j \in \{0, 1\}, k \in \{0, 1, ..., n\}. \tag{S5}$$

where case $\#1(T-1)=k$ represents that there are $k$ distinct edges between the correlation network $N_{T-1}$ and its preceding $N_{T-2}$, or equivalently, there are $k$ edges in the differential network $DN_{T-1}$.

- Testing step: In the testing step, calculate $I$-score of the testing sample, i.e., the differential network $o_T = \{DN_T\}$ at a candidate transition point $t = T$ by Eq.(S16) based on both the HMM $\Theta_{T-1}$ and the additional observation $o_T = \{DN_T\}$. If there is a drastic increase of $I$-score, then $T$ is the switching point, at which system is in the pre-disease stage. Otherwise go to Step-1 for next time point.

The detailed algorithm including the training of HMM and the testing of candidate time point for the $I$-score is presented as following A.3.2 and A.3.3.

### A.3.2 Deriving the HMM based on an unsupervised learning procedure

In order to simplify the derivation of the HMM for of an $n$-variable system ($n$-node network) during its progression, a few notations are presented as follows .

- Denote the time variable as $t$. The progression of a system is along the time series $\{1, 2, ..., T-1, T, ...\}$.
- Denote the correlation network at time point $t = T$ as $N_T$.
- Denote the differential network at time point $t = T$ as $DN_T$.
- Denote the temporal-ordering differential network sequence as $\{DN_2, DN_3, ..., DN_T, ...\}$. Note that this sequence starts from $t = 2$, since each differential network $DN_T$ is obtained by comparing adjacent correlation networks $N_{T-1}$ and $N_T$.
- Denote the observed sequence up to time point $T$ as $O = \{o_1, o_2, ..., o_{T-1}, o_T\}$, where $o_T$ represents the sample set derived at time point $t = T$. Obviously, the temporal-ordering

differential network sequence is obtained based on the observed sequence. Thus we also denote $O = \{o_1, o_2, ..., o_{T-1}, o_T\} = \{DN_2, DN_3, ..., DN_{T-1}, DN_T, ...\}$.

- Denote the state sequence up to time point $t$ as $\{s_1, s_2, ..., s_{t-1}, s_t\}$, i.e., the state of the system is $s_t$ at time point $t$, or equivalently, $s_t = State(o_t)$.
- Denote the unobserved (hidden) states as $W_0$ and $W_1$, where $W_0$ represents the system state before the transition point, and $W_1$ stands for any system state that is not identical with $W_0$, i.e., the state not in the before-transition stage. Therefore, each $s_i$ from the state sequence could be either $s_t = W_0$ if the system is at the before-transition stage at time $t$, or $s_t = W_1$ if the system is not any more at the before-transition stage at time $t$.
- For given HMM $\Theta$ and observation $O$, denote $\gamma_t(i)$ as the probability of the system being at state $W_i$ at time $t$, i.e.,

$$\gamma_t(i) = P(s_t = W_i \,|\, O,\, \Theta) = \frac{P(s_t = W_i,\, O \,|\, \Theta)}{P(O \,|\, \Theta)} \tag{S6}$$

  where $i \in \{0, 1\}$.
- For given HMM $\Theta$ and observation $O$, denote $\xi_t(i, j)$ as the probability of the system being at state $W_i$ at time $t - 1$ and being at state $W_j$ at time $t$, i.e.,

$$\xi_t(i, j) = P(s_{t-1} = W_i, s_t = W_j \,|\, O,\, \Theta) = \frac{P(s_{t-1} = W_i, s_t = W_j,\, O \,|\, \Theta)}{P(O \,|\, \Theta)} \tag{S7}$$

  where $i, j \in \{0, 1\}$.

Following an unsupervised learning scheme, we train a hidden Markov model (HMM) $\Theta_{T-1} = (A, B, \pi)$ based on an observed sequence or differential network series $\{o_1, o_2, ..., o_{T-1}\} = \{DN_2, DN_3, ..., DN_{T-1}, ...\}$, i.e., the first $T-1$ sets of samples from time points $1, 2, ..., T-1$, or equivalently, the preceding $T-2$ differential networks, where the subscript $T-1$ of $\Theta$ represents that the HMM is derived from the training samples up to time point $T-1$, $A$ is a state

transition matrix, $B$ is an emission matrix, and $\pi$ is a probability vector for the initial state. The training process based on an unsupervised learning procedure, namely, Baum-Welch algorithm, is provided as the following four steps.

**Step 1. Construct the differential network at** $(T-1)$**.**

Based on the observed time-course data $O = \{o_1, o_2, ..., o_{T-1}, o_T\}$, we construct the temporal-ordering differential network series for training. The construction of the differential network is as follows (Fig. 1 in the main text).

First we build the correlation network. The correlation network $N_T$ was constructed at each sampling time point $t = T$ (Fig. 1). Each edge connecting two nodes represents the correlation/association between two biomolecules, while each edge connecting only one node represents the self-regulation or variation of the biomolecule. Subsequently, there is a parameter $\alpha$ such that only those edges with high Pearson's correlation coefficients (PCC) ($|PCC| \geq \alpha$) or high standard deviation (SD) ($\frac{SD - min(SD)}{max(SD) - mean(SD)} \geq \alpha$) were reserved, where $\alpha$ is a to-be-determined parameter based on specific real data. Generally, the selection of parameter $\alpha$ is mainly from the computational consideration, that is, $\alpha$ is set such that there are few edges (i.e., significantly changed associations) in the differential networks during the normal stage, thus highlighting the pre-disease stage when many edges arising in a differential network. Specifically, when applied to real data, we choose the cutoff parameter $\alpha$ so that there are only 10% edges in the first differential network (generated from the first and second sampling time points, both of which are assumed to belongs to the normal stage) comparing with original STRING network, that is, over 90% edges disappear comparing with the original STRING network due to the generic property that the network structure would remain stable during the normal stage, and thus there are few edges in a differential network based on samples generated from normal stage.

Second we building the specific network. Comparing the correlation networks $N_T$ and $N_{T-1}$, and the edges which differed between $N_T$ and $N_{T-1}$ were then identified and employed to illustrate the dynamic changes in associations between the adjacent time points $T$ and $T-1$. Then we obtained the specific networks $SN_T = \{N_T\} \setminus \{N_{T-1}\}$ and $SN_{T-1} = \{N_{T-1}\} \setminus \{N_T\}$ respectively by removing the common edges (see Fig. 1d), where the common edges are presented in both correlation networks $N_T$ and $N_{T-1}$ and thus were regarded as interactions without significant change in dynamics.

Third we build the differential network. Combining the two specific networks $SN_T$ and $SN_{T-1}$, i.e., $\{SN_T\} \cup \{SN_{T-1}\}$, we obtained the differential network $DN_T$ at time point $T$, in which the edge connecting two nodes records temporal differential correlation, and the edge connecting only one node records temporal differential variance.

**Step 2. Determine the indicator vector for each variable at $t = T-1$.**

For an $n$-nodeww s differential network $DN(t)$, there could be maximumly $M = \frac{n(n+1)}{2}$ differential edges, including $\frac{n(n-1)}{2}$ differential regulations between two molecules and $n$ self-regulations. We employ an $M$ dimensional vector $E(T-1) = \{e_1(T-1), e_2(T-1), ..., e_M(T-1)\}$ with each variable $e_i$ is an indicator denoting the existence of one certain edge, that is,

$$e_i(T-1) = \begin{cases} 0, & \text{if differential edge does not exist at t} = \mathrm{T}-1 \\ 1, & \text{if differential edge exist at t} = \mathrm{T}-1 \end{cases} . \tag{S8}$$

Therefore, vector $E(T-1)$ records the distribution of the differential edges in a differential network at time $t = T-1$. Obviously, $e_i(T-1) = 1$ represents that an edge is significantly expressed in a differential network $DN_{T-1}$.

According to above settings, we actually transform the differential network $DN(T-1)$ into the corresponding indicator vector $E_{T-1} = \{e_1(T-1), e_2(T-1), ..., e_M(T-1)\}$.

Let $\#0(T-1)$ and $\#1(T-1)$ respectively denote the number of value 0 and that of value 1 in the indicator vector $E(T-1)$ at $T-1$. Obviously, $\#0(t-1) + \#1(t-1) = M$,

where $M$ is the number of all possible edges in a differential network, among which there are $\#0(t-1)$ edges without significant expressions (correlations) in $DN_{T-1}$, while $\#1(T-1)$ edges with significant expressions (correlations) in $DN_{T-1}$. Reminding that the differential network $DN_{T-1}$ is from the comparison of $N_{T-1}$ and $N_{T-2}$, $\#1(T-1)$ represents the number of distinct edges comparing $N_{T-1}$ with $N_{T-2}$, while $\#0(T-1)$ represents the number of identical edges comparing $N_{T-1}$ with $N_{T-2}$.

**Step 3. Training the HMM at $t = T-1$ based on Baum-Welch algorithm.**

In this step, we need to identify the state transition matrix $A$ and the emission matrix $B$ at $(T-1)$, that is, training the HMM $\Theta_{T-1} = (A(T-1), B(T-1), \pi)$ on the basis of an observed sequence $\{o_1, o_2, ..., o_{T-1}\}$, or equivalently, a differential network sequence $\{DN_2, DN_3, ..., DN_{T-1}, ...\}$.

There are two possible states $W_0$ and $W_1$ in time point $t-1$. Then, we calculate the possibilities for each possible state transition and thus obtain the state $A(t-1) = (a_{ij}(t-1))_{2\times 2}$, where

$$a_{ij}(T-1) = P(s_{T-1} = W_i \mid s_{T-2} = W_j), \tag{S9}$$

with $i, j \in \{0, 1\}$.

Besides, for the emission matrix $B(T-1) = (b_{jk}(T-1))_{2\times(n+1)}$ where $b_{jk}(T-1)$ is the probability of the $k$th possible observation under the assumption that the system state is $W_j$ at time $t = T-1$, i.e.,

$$b_{jk}(T-1) = P(\#1(T-1) = k \mid s_{T-1} = W_j), \tag{S10}$$

where $j \in \{0, 1\}$ and $k \in \{0, 1, 2, ..., M\}$, $M$ stands for the number of possible edges in a differential network $DN(T-1)$. Obviously, there are $M$ possible observable cases for any sample at $T-1$, i.e., case $\#1(T-1) = 0$, case $\#1(T-1) = 1$, ... , case $\#1(T-1) = M$. In the case of an $n$-molecules biological network system with $M$ possible edges, case $\#1(T-1) = k$

reflects that there are $k$ edges differentially expressed (i.e., with significant correlations) in one observation $DN_{T-1}$, or equivalently, $\#1(T-1) = k$ distinct edges between $N_{T-1}$ at $t = T-1$ and its former correlation network $N_{T-2}$ at $t = T-2$.

The initial state distribution $\pi = \{\pi_1, \pi_2\}$ is defined at time $T-2$, where

$$\pi_i = P(s_{T-2} = W_i), \tag{S11}$$

with $i \in \{0, 1\}$.

According to Baum-Welch algorithm, we build $A$, $B$, and $\pi$ based on the training set $\{DN_2, DN_3, ..., DN_{T-1}\}$, i.e., differential networks up to time $T-1$. The training process at time $t = T-1$ includes the following three steps.

- **Initialization**

  For $h = 0$, set initial values for $a_{ij}^0$, $b_{jk}^0$, and $\pi_i^0$, we have the HMM $\Theta^0 = (A^0, B^0, \pi^0)$.

- **Update**

  For $h = 1, 2, ...$, we have the update for $a_{ij}^h$, $b_{jk}^h$, and $\pi_i^h$ by recursion

$$a_{ij}^h = \frac{\sum_{t=1}^{T-1} \xi_t(i,j)}{\sum_{t=1}^{T-1} \gamma_t(i)}, \tag{S12}$$

$$b_{jk}^h = \frac{\sum_{t=1, \#1(t-1)=k}^{T-1} \gamma_t(k)}{\sum_{t=1}^{T-1} \gamma_t(k)}, \tag{S13}$$

$$\pi_i^h = \gamma_1(i), \tag{S14}$$

  where $\gamma_t(i)$ and $\xi_t(i,j)$ are respectively of form Eq.(S6) and Eq.(S7). For $\gamma_t(i)$ and $\xi_t(i,j)$, the HMM used in the prior knowledge is that updated from the preceding step.

The observation sequence used in the prior knowledge is $O = \{o_1, o_2, ..., o_{T-1}\} = \{DN_2, DN_3, ..., DN_{T-1}, ...\}$.

- **Ending**

When $h = H$, i.e., the $H$th-updating step, the recursion is terminated. Then

$$\Theta_i^H = (A^H, B^H, \pi^H). \quad \text{(S15)}$$

The HMM used in the testing process is represented as $\Theta_{T-1} = \Theta_i^H$.

#### A.3.3 Calculating the inconsistency score at a testing time point $t = T$

Under the assumption that the transition point is at $t = T$, or in other word, time point $t = T$ is hypothesized as the end point of a stationary Markov process of the before-transition stage. The onset of a pre-transition stage is the end of the stationary Markov process in a before-transition stage.

At each time point $t = T$, we are in a position to test if time $t = T$ is not any more in the before-transition stage, that is, the time point $t = T$ is the switching point for the stationary Markov process described as HMM $\Theta_{T-1}$.

Therefore, at the testing time point $t = T$, we calculate the $I$-index, or HMM-based probability $P_T$ measuring the inconsistence between sample $\{o_T\}$ derived at time point $t = T$ and the HMM $\Theta_{T-1}$, is given as follows:

$$I(T) = P_T(s_T = W_1 \,|\, s_1 = W_0, s_2 = W_0, ..., s_{T-1} = W_0,\, \Theta_{T-1},\, O), \quad \text{(S16)}$$

where the observation sequence $O_T = \{DN_2, ..., DN_{t-1}, DN_T\}$ is the temporal differential networks. Obviously,

$$P_t(s_T = W_1 \,|\, s_1 = W_0, s_2 = W_0, ..., s_{T-1} = W_0,\, \Theta_{T-1},\, O)$$
$$= 1 - Q_t(s_T = W_0 \,|\, s_1 = W_0, s_2 = W_0, ..., s_{T-1} = W_0,\, \Theta_{T-1},\, O).$$

where $Q_T$ actually represents the probability of consistence between the system state of sample $\{o_T\}$ derived at time point $T$ and the HMM $\Theta_{T-1}$. At each time point $t$, we first calculate the consistence probability based on HMM $\Theta_{T-1}$ and observed sequence $O = \{o_{T-1}, o_T\}$

$$Q_T(s_T = W_0 \mid s_{T-1} = W_0,\, \Theta_{T-1},\, O) = \frac{P(s_{T-1} = W_0, s_T = W_0 \mid \Theta_{T-1},\, O)}{P(s_{T-1} = W_0 \mid \Theta_{T-1},\, O)}. \tag{S17}$$

The numerator

$$P(s_{T-1} = W_0, s_T = W_0 \mid \Theta_{T-1},\, O) = \frac{\beta_{T-1}(s_{T-1} = W_0)a_{00}b_{0k}}{\sum_{i=0}^{1} \beta_{T-1}(s_{T-1} = W_i)a_{ij}b_{jk}}, \tag{S18}$$

and the denominator

$$P(s_{T-1} = W_0 \mid \Theta_{T-1},\, O) = \frac{\beta_{T-1}(s_{T-1} = W_0)}{\sum_{j=0}^{1} \beta_{T-1}(s_{T-1} = W_j)}, \tag{S19}$$

where $a_{00}$ and $a_{ij}$ is from the state transition matrix $A = (a_{ij})_{2\times 2}$ in Eq.(S4), $b_{0k}$ and $b_{jk}$ is from the emission matrix $B = (b_{jk})_{2\times(n+1)}$ in Eq.(S5) while $k = \#1(T)$ represents that for the differential network $DN_T$ there are $k$ edges differentially expressed, $\beta$ is the forward probability provided as below Eq.(S20). It should be noticed that in Eqs.(S18) and (S19) the backward probability is set to be 1, since differential networks $DN_{T+1}, \cdots$ are not available when $t = T$ is the testing time point.

According to above settings, given the HMM $\Theta_{T-1}$, the calculation of HMM probability $Q_T$ (the consistency probability) at a time point $t = T$ only relies on the samples from $T-1$ and $T$. Obtaining the HMM probability $Q_T$ for every candidate time point, the time point $arg_t[min(Q_t)]_{t=1,2,...,T}$, or equivalently $arg_t[max(P_t)]_{t=1,2,...,T}$, is the transition point.

**The HMM-based forward probability.**

For given observation sequence $\{o_1, o_2, ..., o_T\}$ or equivalently a differential network sequence $\{DN_2, DN_3, ..., DN_{T-1}, DN_T\}$, two possible states $\{W_0, W_1\}$, and HMM $\Theta = (A, B, \pi)$

with $A = (a_{ij})_{2\times 2}$, $B = (b_{jk})_{2\times(n+1)}$ with $b_{jk}$ related to observation $o_T = DN_T$, and $\pi = (\pi_i)_{i\in\{0,1\}}$, we calculate the HMM-based forward probability

$$\beta_T(i) = \beta_T(s_T = W_i) = P(DN_2, DN_3, ..., DN_{T-1}, DN_T, s_T = W_i \,|\, \Theta), \quad i \in \{0, 1\}. \tag{S20}$$

as follows.

**Initialization:** For $t = 1$,

$$\beta_1(i) = \pi_i b_{ik_1}, \quad i = 0, 1, \tag{S21}$$

where $k_1$ is given in $\{1, 2, ..., M + 1\}$.

**Recursion:** For $t = 2, 3, ..., T - 1$

$$\beta_t(i) = \left[\sum_{j=0}^{1} \beta_{t-1}(j) a_{ji}\right] b_{ik_t}, \quad i = 0, 1. \tag{S22}$$

where $k_t = \#1(t - 1)$ with $k_t \in \{1, 2, ..., n + 1\}$.

**Ending:** When $t = T$,

$$\beta_T(0) = \beta_t(s_t = W_0) = \left[\sum_{j=0}^{1} \beta_{T-1}(j) a_{j0}\right] b_{0k_T},$$

$$\beta_T(1) = \beta_t(s_t = W_1) = \left[\sum_{j=0}^{1} \beta_{T-1}(j) a_{j1}\right] b_{1k_T},$$

and

$$P(O \,|\, \Theta) = P(DN_2, DN_3, ..., DN_{T-1}, DN_T \,|\, \Theta) = \sum_{i=0}^{1} \beta_T(i). \tag{S23}$$

# B Numerical validation of $I$-score

In this section, we use a eight-gene network (see Fig. S24 or Fig. 3a in the main text) to conduct a numerical simulation and theoretically demonstrate the detection of early-warning signals through $I$-score scheme. These types of gene regulatory networks are often used to study transcription, translation, diffusion, and translocation processes that affect gene regulatory

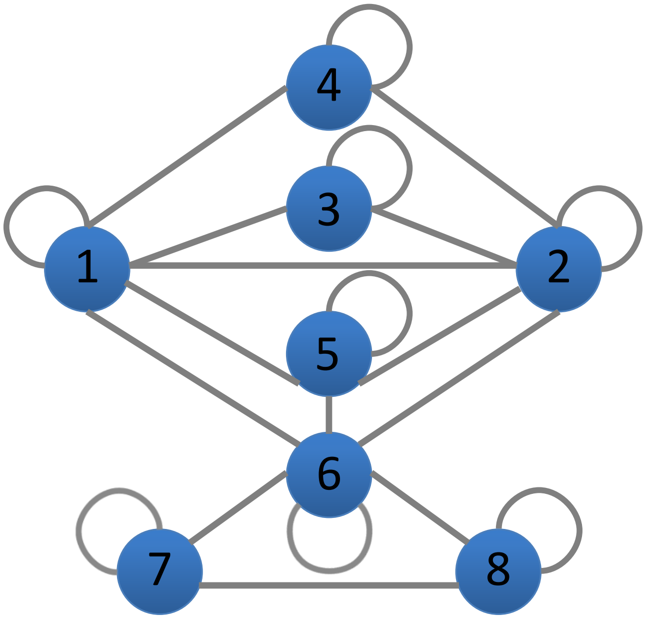


Figure S1: | **A model of an eight-molecular network.** In this sketch of a molecular network, there are eight nodes whose dynamical regulatory relationships are given as stochastic system S24. The edges represent positive or negative regulations among nodes.

activities (*1, 4, 19–21*). The following six differential equations represent the gene regulation of six genes in a network where gene regulation is represented in a Michaelis-Menten form, with the exception of the degradation rates, which are linearly proportional to the concentrations of the corresponding genes.

$$\left\{\begin{array}{l}
\frac{\mathrm{d}z_1(t)}{\mathrm{d}t} = \frac{(8-4\,|q|)z_2(t)}{15\,(1+z_2(t))} - \frac{4+4\,|q|}{15}\,z_1(t) + \zeta_1(t),\\
\frac{\mathrm{d}z_2(t)}{\mathrm{d}t} = \frac{(4-2\,|q|)z_1(t)}{15\,(1+z_1(t))} - \frac{8+2\,|q|}{15}\,z_2(t) + \zeta_2(t),\\
\frac{\mathrm{d}z_3(t)}{\mathrm{d}t} = \frac{4\,|q|-10}{15} + \frac{5-2\,|q|}{15(1+z_1(t))} + \frac{5-2\,|q|}{15(1+z_2(t))} - z_3(t) + \zeta_3(t),\\
\frac{\mathrm{d}z_4(t)}{\mathrm{d}t} = \frac{4\,|q|-12}{15} + \frac{(6-2\,|q|)z_1(t)}{15(1+z_1(t))} + \frac{(6-2\,|q|)z_2(t)}{15(1+z_2(t))} - \frac{6}{5}z_4(t) + \zeta_4(t),\\
\frac{\mathrm{d}z_5(t)}{\mathrm{d}t} = \frac{4\,|q|-14}{15} + \frac{(7-2\,|q|)z_1(t)}{15(1+z_1(t))} + \frac{(7-2\,|q|)z_2(t)}{15(1+z_2(t))} - \frac{7}{5}\,z_5(t) + \zeta_5(t),\\
\frac{\mathrm{d}z_6(t)}{\mathrm{d}t} = -\frac{11}{15} + \frac{1}{15(1+z_1(t))} + \frac{1}{15(1+z_2(t))} + \frac{z_3(t)}{5(1+z_3(t))} + \frac{1}{5(1+z_5(t))} + \frac{1}{5(1+z_7(t))}\\
\qquad + \frac{1}{5(1+z_8(t))} - \frac{8}{5}\,z_6(t) + \zeta_6(t),\\
\frac{\mathrm{d}z_7(t)}{\mathrm{d}t} = \frac{z_8(t)}{10(1+z_8(t))} - \frac{19}{10}\,z_7(t) + \zeta_7(t),\\
\frac{\mathrm{d}z_8(t)}{\mathrm{d}t} = \frac{z_7(t)}{10(1+z_7(t))} - \frac{19}{10}\,z_8(t) + \zeta_8(t),
\end{array}\right. \tag{S24}$$

where $P$ is a scalar control parameter and $\zeta_i(t)$ $(i = 1, 2, ..., 10)$ are Gaussian noises with zero means and covariances $\kappa_{ij} = \mathrm{Cov}(\zeta_i, \zeta_j)$. $z_i$ $(i = 1, ..., 8)$ represent the concentrations of mRNA-$i$. In Eq.(S24), the degradation rates of mRNAs are $(\frac{4+4|q|}{15}, \frac{8+2|q|}{15}, 1, \frac{6}{5}, \frac{7}{5}, \frac{8}{5}, \frac{19}{10}, \frac{19}{10})$. There is a stable equilibrium point $\bar{Z} = (\bar{z}_1, \bar{z}_2, ..., \bar{z}_8) = (0, 0, 0, 0, 0, 0, 0, 0)$. The differential equations Eq.(S24) can be transformed into the difference equations $Z(k+1) = f(Z(k), P)$ using the Euler scheme (*22*), *i.e.,*

$$\begin{cases} z_1(k+1) = z_1(k) + \left[\frac{(8-4\,|q|)z_2(k)}{15\,(1+z_2(k))} - \frac{4+4\,|q|}{15}\,z_1(k) + \zeta_1(k)\right]\Delta t, \\ z_2(k+1) = z_2(k) + \left[\frac{(4-2\,|q|)z_1(t)}{15\,(1+z_1(k))} - \frac{8+2\,|q|}{15}\,z_2(k) + \zeta_2(t)\right]\Delta t, \\ z_3(k+1) = z_3(k) + \left[\frac{4\,|q|-10}{15} + \frac{5-2\,|q|}{15(1+z_1(k))} + \frac{5-2\,|q|}{15(1+z_2(k))} - z_3(t) + \zeta_3(k)\right]\Delta t, \\ z_4(k+1) = z_4(k) + \left[\frac{4\,|q|-12}{15} + \frac{(6-2\,|q|)z_1(k)}{15(1+z_1(k))} + \frac{(6-2\,|q|)z_2(k)}{15(1+z_2(k))} - \frac{6}{5}z_4(k) + \zeta_4(k)\right]\Delta t, \\ z_5(k+1) = z_5(k) + \left[\frac{4\,|q|-14}{15} + \frac{(7-2\,|q|)z_1(k)}{15(1+z_1(k))} + \frac{(7-2\,|q|)z_2(k)}{15(1+z_2(k))} - \frac{7}{5}\,z_5(k) + \zeta_5(k)\right]\Delta t, \\ z_6(k+1) = z_6(k) + \left[-\frac{11}{15} + \frac{1}{15(1+z_1(k))} + \frac{1}{15(1+z_2(k))} + \frac{z_3(k)}{5(1+z_3(k))} + \frac{1}{5(1+z_5(k))} + \frac{1}{5(1+z_7(k))}\right. \\ \qquad\qquad \left. + \frac{1}{5(1+z_8(k))} - \frac{8}{5}\,z_6(k) + \zeta_6(k)\right]\Delta t, \\ z_7(k+1) = z_7(k) + \left[\frac{z_8(k)}{10(1+z_8(k))} - \frac{19}{10}\,z_7(k) + \zeta_7(k)\right]\Delta t, \\ z_8(k+1) = z_8(k) + \left[\frac{z_7(k)}{10(1+z_7(k))} - \frac{19}{10}\,z_8(k) + \zeta_8(k),\right]\Delta t, \end{cases} \tag{S25}$$

with a small time interval $\Delta t$. Note that $Z(k)$ is the vector of $Z(t)$ at the time instant $k\Delta t$.

We denote the Jacobian matrix of Eq.(S25) as $J = \left.\frac{\partial f(Z(k);p)}{\partial Z}\right|_{Z=\bar{Z}}$, where

$$J = \mathrm{e}^{\Delta t \cdot A} \tag{S26}$$

with $A$ is the coefficient matrix of the linearized system of Eq.(S24).

From Eq.(S26), we obtain eight distinct eigenvalues $(0.67^{|P|}, 0.45, 0.37, 0.30, 0.25, 0.20, 0.17, 0.14)$ by taking $\Delta t = 1$. It is obvious that the largest eigenvalue $0.67^{|P|} \to 1$ when $P \to 0$. Thus there is a bifurcation for the diffence system Eq.(S25). Thus, the equilibrium point $\bar{Z}$ is stable when $P \in (-1, 0]$. Obviously, there is a critical value $P_c = 0$, where the system loses stability and undergoes a critical transition. We aimed to detect early warning signals that indicate the critical transition as a control parameter $P$ approaches a critical value 0 from $P < 0$.

Based on the simulated model, we collected time-course data of the eight-node expressions. Then, based on these simulated data we calculated the inconsistency score curves for the network, as shown in Fig. 3 in the main text.

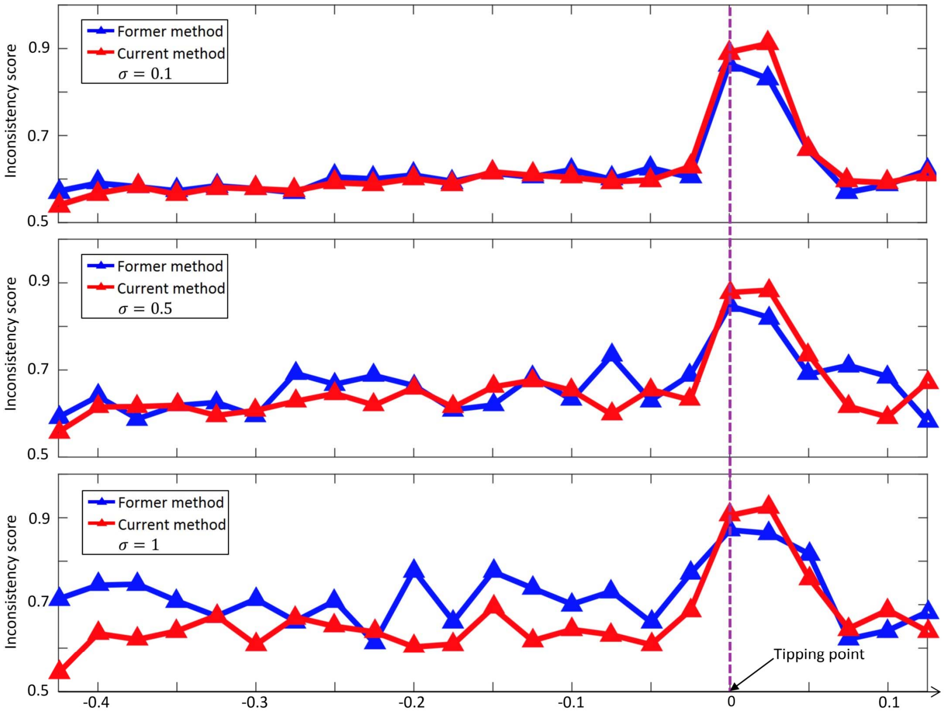


Figure S2: | **Comparison of the performance between current and former approaches.** To compare the performance between current method and former approach proposed in (*24*), these two methods are respectively applied to a simulation dataset generated by the 8-nodes artificial network in Fig. S1 under different noises. Aiming to signal the tipping point at $q = 0$, it can be seen that under small noise ($\sigma = 0.1$), there is no significant difference. However, when under large noise ($\sigma = 0.5$, or $\sigma = 1$), the current method is more robust and stable. This is because that in current method, not only the nodes but the associations among nodes are considered while in the former approach, only nodes are taken into consideration.

It is worth noting that the proposed algorithm is much more robust in detecting the tipping point during the biological processes than the former one proposed in (*24*). Actually, the differential network based algorithm is an effective and robust aggregate method for exploiting the dynamical information from the longitudinal measurements of molecular factors involved in a disease, and better describe the abrupt dynamical change. An illustrative figure shows the

different performance between the proposed algorithm and that in the former published paper as Fig. S2. To compare the performance between current method and former approach proposed in (*24*), the two methods are respectively applied to a simulation dataset generated by the 8-nodes artificial network (Fig. S1) under different noises. Aiming to signal the tipping point at $q = 0$, it can be seen that under small noise ($\sigma = 0.1$), there is no significant difference. However, when under large noise ($\sigma = 0.5$, or $\sigma = 1$), the current method is more robust and stable. This is because that in current method, not only the nodes but the associations among nodes are considered while in the former approach, only nodes are taken into consideration.

# C Application to three real datasets

For the three datasets, that is, gene expression profiling dataset of acute lung injury induced by phosgene gas (access ID: GSE2565), the microarray dataset of stimulating-stress production caused by acute corneal trauma (access ID: GSE1393), and genomic data of MCF-7 cell line human breast cancer caused by heregulin (access ID: GSE13009), we identified the pre-transition states through $I$-score.

The gene expression profiling dataset of the three diseases was downloaded from the NCBI GEO database (www.ncbi.nlm.nih.gov/geo). In these datasets, probe sets without corresponding gene symbols were ignored during our analysis. The expression values of probe sets that are mapped to the same gene were averaged. The three datasets are described in Table S1. Besides, we described the data processing in details and conducted the functional analysis results (g:profiler: http://biit.cs.ut.ee/gprofiler/ and NOA: http://app.aporc.org/NOA/) (*25,26*) for some important molecules.

Table S1: Descriptions of the three datasets

| Experimental data | Description |
|---|---|
| Genomic data of lung injury due to carbonyl chloride inhalation exposure (GSE2565) (*29*) | |
| Sampling points | 9 sampling points<br>0, 0.5, 1, 4, 8, 12, 24, 48, and 72 (hours) |
| Number of observed objects | 22690 genes |
| Groups | control group and case group |
| Case data | 6 subjects |
| Control data | 6 control samples |
| Genomic data of MCF-7 human breast cancer caused by heregulin (HRG) (GSE13009) (*31*) | |
| Sampling points | 16 sampling points<br>1/4, 1/3, 1/2, 3/4, 1,<br>3/2, 2, 3, 4, 6, 8, 12,<br>24, 36, 48, 72 (hours) |
| Number of observations | 13785 genes |
| Groups | control group and case group |
| Case data | 2 subjects |
| Control data | 3 control samples |
| Genomic data on stimulating-stress production by acute corneal trauma (GSE1393) (*32*) | |
| Sampling points | 8 sampling periods<br>0.5, 1, 3, 8, 24, 72, 120, and 360 (hours) |
| Number of observations | 15,065 genes |
| Groups | control group and case group |
| Case data | 5-6 replicates |
| Control data | 5-6 replicates |

## C.1 Dataset 1. Genomic data of the lung injury with carbonyl chloride inhalation exposure (i.e., acute lung injury)

This dataset was obtained in an experiment on toxic gas-induced lung injury effects, i.e., pulmonary edema (*29*), and was downloaded from the NCBI GEO database (ID: GSE2565) (www.ncbi.nlm.nih.gov/geo). In this dataset, probe sets without corresponding gene symbols were not considered during our analysis. The expression values of probe sets mapped to the same gene were averaged. Genes for the this disease have been linked and correlated by the combined functional couplings among them from various databases of protein-protein interac-

tions of STRING and BioGrid. In the disease dataset, the expression profiling information was mapped to the integrated networks individually for constructing the correlation networks. We downloaded the biomolecular interaction networks from various databases, including STRING (http://string-db.org/), BioGrid (www.thebiogrid.org), KEGG (www.genome.jp/kegg), and H-PRD (www.hprd.org). First, the available functional linkage information for *Mus musculus* was downloaded from these databases and combined. For instance, after removing the redundancy, we obtained 37950 linkages in 6683 mouse proteins/genes for acute lung injury. Next, the genes evaluated in these microarray datasets were mapped individually to these integrated functional linkage networks. The network information are employed in the post processing step for visualizing results (Figs.3a, 4a,4b in the main text) and functional analysis.

To study acute lung injury, a genomic approach was used to investigate the molecular mechanism of phosgene-induced lung injury. The experiments were conducted to determine the temporal effects of phosgene exposure on lung tissue antioxidant enzyme concentrations and the gene expression level, and these results were compared with those from air-exposed mice treated in a similar manner to assess the role of the GSH redox cycle in this oxidative lung injury model. To produce two groups of data, i.e., the control group data and case group data, two groups of CD-1 male mice were exposed to air or phosgene, respectively. Lung tissues were collected from air- or phosgene-exposed mice at 0.5, 1, 4, 8, 12, 24, 48, and 72 hours (h) after exposure. The details of the experiment are available in the original paper (*29*). We introduce some key background as well as our functional analysis as follows.

Phosgene gas is one of the most important and common chemical industry gases (*28*). Some pathogenic mechanisms of the acute lung injury induced by phosgene have been identified (*29*). Dysfunctions of glutathione metabolism and the chemokine signaling pathway related to inflammatory immune response were activated *in vivo*, which also reflected protection against the oxidant-like activity of phosgene. Pathways affected by the oxidant reaction became dis-

ordered, especially signal transduction via protein-modified activation, such as the MAPK signaling pathway and Wnt signaling pathway. The decrease in pH induced by the HCl-release reaction affected some pathways that were sensitive to intracellular conditions and related to communication or transport channels, e.g., gap junctions. Some signaling pathways may also be relevant to repair, survival, apoptosis, and reproduction, such as the GnRH signaling pathway, MAPK signaling pathway, and TGF-beta signaling pathway (*29, 30*). At the GO function level, some biological processes were also highly related to acute lung injury. For example, the expression profiles of some genes were related to abnormal changes in primary metabolic processes. This indicated the denaturation of lipoids, proteins, and nucleic acids that may have been oxidized by phosgene (*29, 30*). Some well-known genes that regulate or are directly involved in apoptosis were also included in the local networks with significant $I$-scores, such as *JUN*, *NOTCH2*, and *MYC*. Some genes in the 10% top differential genes selected from the local networks with significant $I$-scores were also related to inflammatory response, wounding induced by oxidant damage, and irritation, such as *IL1B*, *PTGS2*, *CCL2*, and *MYD88*. Some local networks are presented in the Fig. 4 in the main text.

Briefly, investigators found that the main physiological effects occurred within the first 8 hours after exposure, resulting in common observations of enhanced BALF protein levels, increased pulmonary edema, and ultimately decreased survival rates (*29*). At the concentration delivered, 50%-60% mortality was routinely observed at 12 hours while 60%-70% mortality was observed at 24 hours (*29*). The detailed results are also available in the original paper (*29*). Early warning signals of lung injury based on the $I$-score are shown in Fig. 3 of the main text, which showed that the pre-transition state may start around 4 h, while the system may enter the after-transition state after 12 h. Our prediction based on the $I$-scores agreed with the actual disease development.

To explain the how the $I$-score scheme applied in real dataset more clearly, we used acute

lung injury as a concrete example to describe our computational procedure step by step. In GSE2565 data set, there are 22,690 original probe sets. We mapped them to the corresponding NCBI Entrez gene symbols by using the GEO annotation. Meanwhile, we screened out all probe sets with incorrect corresponding gene symbols while probe sets that detected the same genes were combined using the averaging method. After this procedure, there were 12,871 genes left.

Step 1 Choose differential expression genes from the high-throughput gene data for acute lung injury. Then construct the temporal-ordering differential network sequence. At each sampling point (or period), there are 12,871 genes. Each gene has 6 case samples and 6 control samples. At the 0 h sampling point, the case samples are identical to the control samples. Each sampling time point is supposed as a candidate tipping point of the critical transition, based on which we apply the $I$-score algorithm and calculate the inconsistency score representing the probability of a candidate point being the end point of the stationary Markov process of the before-transition state.

At each sampling point, by the student t-test with statistical significance ($p < 0.05$) and fold change, the genes are filtered. The numbers of differentially-expressed gene are $\{503, 840, 1635, 1892, 1393, 1337, 1620, 1399\}$ respectively at 0.5, 1, 4, 8, 12, 24, 48 and 72 hours (h).

According to the scheme of inconsistency score, except the first sampling time point (0 h) which is always supposed as in the before-transition stage, we regard that each time point is a candidate critical point, i.e., the end point of the stationary Markov process in a before-transition stage. For each candidate critical point $t = T$, the differentially-expressed genes selected at this time point are mapped to STRING network (http://string-db.org/), based on which the correlation network $N_T$ is constructed

by a threshold scheme, that is, for edges connecting with two nodes (the correlation between two biomolecules), only those with high correlation coefficients are reserved; while for edges connecting with only one node (the self-regulation or the variation of a biomolecule), only those with high standard variation are reserved. Subsequently, comparing the adjacent correlation networks, i.e., $N_T$ and $N_{T-1}$, we obtain the differential network $DN_T = N_T - N_{T-1}$ by removing the common edges.

Step 2 As the training step, we carried out the $I$-score scheme and the Baum-Welch algorithm.

We conducted the data normalization for all the variables.

$$A = \frac{D_{\text{case}} - \text{mean}(N_{\text{control}})}{\text{SD}(N_{\text{control}})}, \tag{S27}$$

where $A$ denotes the normalized expression data for each variable in each case sample, $D_{\text{case}}$ is the data for each variable in every case sample, while the mean($N_{\text{control}}$) and SD($N_{\text{control}}$) are the mean and standard deviation for each variable in all the control samples, respectively.

Regard the differential networks $\{DN_t(t)\}, (t = 0.5, 1, ..., 72)$ (where samples in $t = 0$ are set as reference) as the observation sequence. From the second sampling time point, at a candidate critical time point $T$, we trained the HMM at $T - 1$ following the above algorithm.

Step 3 As the testing process, we calculated the $I$-score via the forwarding algorithm.

Step 4 The sharp increase of the $I$-score indicates the impending critical transition and the peak of the $I$-score demonstrates the most possible tipping point of the critical transition.

The obtained inconsistency score curve is consistent with the real experimental phenomenon, and the inconsistency score start increasing sharply from the 4th time period (4 h) and reach

peaks in the 5th time period (8 h, i.e., the pre-transition, see Fig. 3a in the main text). This indices show that the pre-transition state starts near the 4th time period (4 h), and the system transitions to another state after the 5th time period (8 h). Our early-warning signals are coincident with the actual disease development that the most prominent physiological effects occur within the first 8 h after exposure, resulting in pulmonary edema and ultimately reducing survival rates (see the original paper (*29*)). Based on the dynamical information of the network, we have graphically illustrated the dynamical changes in the overall mouse PPI network (see Fig. 4b in the main text), from which it can be seen that a critical transition occurs around 8 h sampling point (during 4-8 h period).

For the specific algorithm for this real dataset, we have the following three notations.

**Note 1** During the progression of the disease, each time point is supposed as a candidate transition point. To validate a candidate point, we tested whether some genes show significant changes and behave dynamically in a strongly collective manner, i.e., the distribution of these genes change drastically comparing with their previous distribution. Therefore, we respectively investigate the set of genes differentially expressed at each candidate point, rather than the whole set of differential genes identified from all the time points.

**Note 2** To compare the $I$-score, it is required that the number of the nodes in the testing differential networks is equal at all candidate time points. Specifically, in the HMM-training step at a candidate time point, the emission matrix $B$ is $2 \times (n + 1)$ dimension, where $n$ denotes the number of variables. To unify the scale of the $I$-score, so that the inconsistency indices from different candidate time points are comparable, we only consider the same number of genes in the testing differential network at each candidate point.

**Note 3** Generally, it is a heavy burden of computation to calculate the $I$-score by employing the whole differential network, since if there are $n$ nodes in a network, there could be

at most $\frac{n(n+1)}{2}$ differential edges, including $\frac{n(n-1)}{2}$ differential regulations between two molecules and $n$ self-regulations. Thus there could be up to $2^{\frac{n(n+1)}{2}}$ cases in the emission matrix. To reduce the computational complexity, the differential network is partitioned into many local networks. Each local network contains a center node (a differentially-expressed gene) and all of its first-order neighbours based on the network structure, such as a integrated STRING network. The $I$-score for each local network is then calculated piece by piece and thus generate an weighted average score. Specifically, suppose there are $k$ subnetworks partitioned from a differential network,

$$I = \frac{n_1 I_1 + n_2 I_2 + \cdots + n_k I_k}{n_1 + n_2 + \cdots + n_k}, \tag{S28}$$

where $n_i$ denotes the number of nodes in the $i$-th local network and $I_i$ stands for the local $I$-score of this subnetwork.

**Note 4** Clearly, for different testing curve based on distinct candidate critical point, the testing differential network contains distinct genes and structure. For example, in Fig. 3a of main text, the red curve (the testing curve for the candidate critical point 8 h) is calculated based on the testing differential network obtained at 8 h, which is distinct to the testing network of any blue curve (one of the testing curves for other candidate critical points). However, for one testing curve, say, the red curve, the $I$-score is calculated by using the identical testing differential network, i.e., the differential network obtained at 8 h.

To better show the significance and consistence of our results, we have plotted the inconsistency scores generated from bootstrap as in Fig. S3. Carrying out leave-one-out scheme, we obtain 6 sets of samples at each time point, based on which we respectively calculated the inconsistency scores. From Fig. S3, it can be seen that the $I$-score curves based on the re-sampled datasets all show significant signals to the critical transition at or around 8 h.

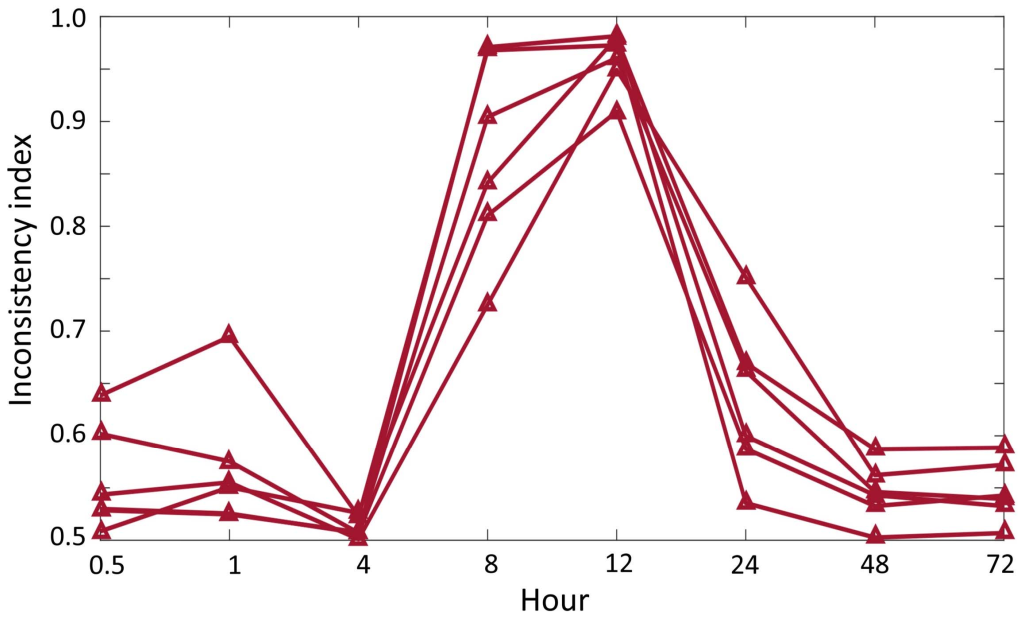


Figure S3: | **The inconsistence indices generated from leave-one-out bootstrap.** To illustrate the significance of the results, the inconsistency score is calculated respectively based on 6 sets of datasets generated from leave-one-out bootstrap. It can be seen that the $I$-score curves based on the re-sampled datasets show significant signal to the critical transition around 8 h.

We screened genes in the top significant differential local networks at the identified critical time point (8 h), among which some well-known genes that regulate or are directly involved in apoptosis were also included in the most significant group, such as JUN, NOTCH2, and MY-C. Some genes in the most significant group were also related to the inflammatory response, wounding induced by oxidant damage, and irritation, such as IL1B, PTGS2, CCL2, and MY-D88, and presented the dynamical changes in the network including the selected genes during the progression in the Fig. 3c of the main text.

Twelve local networks are identified with most significant changes in $I$-score around the critical point, which are showed in Fig. 4 of the main text. To analyze and illustrate the dynamical difference before and after the critical transition, graph-related information is shown in the Table 1 in the main text. It can be seen that the structures of these networks changes drastically between normal and disease states. The ratios of the overturn neighbors and edges overwhelm

those of the background 28.6% (overturn neighbors) and 11.7% (overturn edges), i.e., the ratio of the overturn nodes/edges in the whole STRING molecular network.

## C.2 Dataset 2: Genomic data on acute corneal trauma

Corneal injury, or overstimulation, is known to alter gene expression in the lacrimal gland. Earlier experiments identified several growth factors and other genes that were differentially expressed in the lacrimal gland after creation of a corneal epithelial defect. Damage to cornea sensory nerve terminals that creates partial denervation is hypothesized to be the main cause of dry eye syndrome after refractive surgery and normal aging (*33*). Corneal injury, or overstimulation, is known to alter gene expression in the lacrimal gland. The chemical burn not only damages the cornea and its nerves, but also creates an indirect, yet predictable overstimulation of the lacrimal gland through the neuronal circuit between the two organs (*32*). To study the overstimulation and the related gene regulation, a mouse model with acute corneal trauma was created with a chemical burn to the cornea with silver nitrate, based on which the dataset GSE1393 was obtained and studied. In the experiment, sixty-four female BALB/c mice at 12 weeks of age were randomly divided into eight groups, eight mice per group. The corneas of four mice in each group were bilaterally cauterized with silver nitrate, and another four time-matched mice were used as the control. The total RNA of the lacrimal gland was then extracted, at eight time points (0.5, 1, 3, 8, 24, 72, 120, and 360 hours after the corneal burn), and gene expression was examined with using cDNA microarray technology (*32*).

According to our algorithm, the critical transition of the overstimulation after acute corneal trauma occurred around 1-8 h period, due to the drastic increase of $I$-score.

Interestingly, we found that many of genes in the local differential networks with significant $I$-score were consistent with the response to stress response in vivo, particularly the activation of the immune system and dysfunctions associated with basic cell metabolism in the hosts.

Some enriched pathways were related to dysfunctions of a combination of neuronal and hormonal pathways. Some pathways shared common characteristics with cancer, especially the signaling pathways involved in cell growth, such as transcriptional misregulation in cancer, purine metabolism, Wnt signaling pathway, TGF-beta signaling pathway, and others. These dysfunctional pathways indicated the cell status.

In the acute corneal trauma dataset GSE1393, there were 16,928 original probesets, which we mapped to the PPI network. We screened out all probesets with incorrect corresponding gene symbols while probeset that detected the same genes were combined using the averaging method, leaving 10,354 genes. Using the algorithm in Section E, we conducted the computation of $I$-score and get the application result, which suggests that the critical transition is around the 8 h time point.

## C.3 Dataset 3: Genomic data of MCF-7 human breast cancer caused by heregulin (HRG)

Great attention has been paid to the study of breast cancer because it is one of the highest fatal-ratio diseases and increasing numbers of female patients are suffering from breast cancer worldwide. The main cause of breast cancer is far from clear, but some of the mechanisms and phenomena that occur during the disease progression of HRG-induced breast cancer have been identified based on studies of its molecular mechanisms (*31, 34*) and pathogenesis (*35*).

Heregulin (HRG) induces dose-dependent transient and sustained intracellular signaling, proliferation, and differentiation of MCF-7 breast cancer cells. This dataset was obtained in an experiment on MCF-7 cell line with HRG stimulation (GSE13009).

In the infected host, some metabolic pathways respond to these interruptions and become increasingly disordered. The following results show that some reported phenomena were consistent with our investigations, which also provided some novel insights. In order to analysis

the biological function of the genes with significant $I$-score (in the sense of the $I$-score of the local network centered with a gene), we screened some local network and their centers.

The identified local network is related to regulation of apoptotic process (GO:0042981), regulation of programmed cell death (GO:0043067) and regulation of cell death (GO:0010941) with significant P-value of gene enrichment by website tools of DAVID Bioinformatics Resource (*36*). By the pathway analysis in KEGG database, we found that 7 genes (*CEBPA*, *SMAD3*, *GSK3B*, *LAMC2*, *MMP1*, *PIK3R3*, *RXRA*) in these local networks participate in the pathways in cancer, and many genes of this module also take part in the other cancer related pathways, e.g. *Wnt* signaling pathway, *p53* signaling pathway, ECM-receptor interaction. Many genes in these local networks with significant $I$-scores have been proved to associate with cancer or tumor process, and some of these genes are associated with breast cancer. For example, *BCAS4* is an important gene for breast tumor development and progression (*37*), *ARID3B* is one of genes to regulate cell motility and actin cytoskeleton organization (*38*), and is found to associate with the onset of breast cancer (*39*). *TNFRSF21* encodes tumor necrosis factor receptor which can regulate the NF-kappaB and mediate apoptosis process (*40*). *LAMC2* encodes the gamma chain isoform laminin, which is involved in many biological process, and LAMC2 also is proved to be related to breast cancer process (*41, 42*). Therefore, local networks with significant $I$-scores for HRG-induced breast cancer can mainly induce cancer by affecting the processes of regulation of apoptosis, regulation of programmed cell death and regulation of cell death.

Figure S4 presents the second application of inconsistency score for MCF-7 human breast cancer caused by heregulin (HRG). Based on Fig. S4a, each probability curve is calculated based on a testing differential network at the corresponding candidate critical time point. The red curve in Fig. S4a shows the most significant increase of inconsistency probability at the 90-minutes time point, which indicates that the most possible critical transition point is around

90 minutes. Fig. S4b shows the dynamical evolution in the whole feature network based on the case data of breast cancer. It can be seen that the network structure also changes significantly at the 90-minutes time point. In fact, the original assay showed that the AP-1 complex in HRG-treated MCF-7 cells contains c-JUN, c-FOS and FRA-1, although the association of c-JUN in the complex is transient (*31*). Besides, the stimulation of MCF-7 breast cancer cells with EGF and HRG resulted in very similar early transcription profiles up to 90 min; however, subsequent cellular phenotypes differed after 3 h (*31*), which suggests that the differentiation is around 3 h (the 9th sampling time point). Therefore, our application results are in coincidence with the experimental observation and successfully detect the early-warning signal of the impending critical transition.

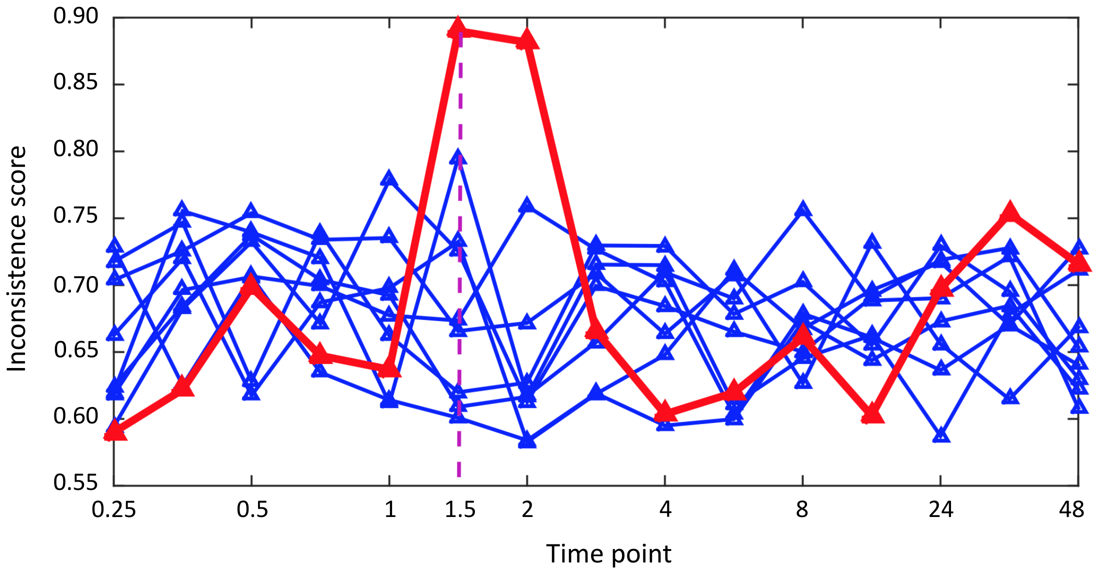


Figure S4: | **The application of $I$-score scheme on the dataset of HRG-induced breast cancer.** By applying $I$-score scheme to the microarray data of HRG-induced breast cancer, we show the $I$-scores from each candidate transition time points. The red curve represents the $I$-score calculated from the testing differential network which is obtained at the 6th sampling point (1.5 h), while the blue curves are that from other time points. It is can be seen that the most significant signal appear around the 1.5 h, which agrees with the experimental observation, that is, the stimulation of MCF-7 breast cancer cells with EGF and HRG resulted in very similar early transcription profiles up to 1.5 h. However, subsequent cellular phenotypes differed after 3 h, which suggests that the differentiation is around 3 h (the 9th sampling time point).

## References


1. Chen, L., Wang, R., Li, C. & Aihara, K. *Modeling Biomolecular Networks in Cells: Structures and Dynamics*, (Springer, New York, 2010).

2. Voit, E.O. A systems-theoretical framework for health and disease: Inflammation and preconditioning from an abstract modeling point of view, *Math. Biosci.* **217**, 11–18(2009).

3. Hovinen, E., Kekki, M. & Kuikka, S. A theory to the stochastic dynamic model building for chronic progressive disease processes with an application to chronic gastritis, *J. Theor. Biol.* **57**, 131–152(1976).

4. Chen, L., Wang, R. & Zhang, X. *Biomolecular Networks: Methods and Applications in Systems Biology*, (John Wiley & Sons, Hoboken, New Jersey, 2009).

5. Guckenheimer, J. & Holmes, P. *Nonlinear Oscillations, Dynamical Systems, and Bifurcations of Vector Fields*, (Springer, 1983).

6. Arnol'd, V.I. *Dynamical systems V: bifurcation theory and catastrophe theory*, (Springer, 1994).

7. Murdock, J. *Normal forms and unfoldings for local dynamical systems*, (Springer, 2003).

8. Wiggins, S. *Global bifurcations and chaos: analytical methods*, (Springer, 1988).

9. Mlodinow, L. *The Drunkard's Walk*, (New York: Random House, 2008).

10. Cover, T. & Thomas, J. *Elements of information theory*, (Wiley, New Jersey, 2005).

11. Strogatz, S. H. *Nonlinear Dynamics And Chaos: With Applications To Physics, Biology, Chemistry And Engineering*, (Addison-Wesley, Reading, MA, 1994).

12. Chen,L. *et al.* (2012) Detecting early-warning signals for sudden deterioration of complex diseases by dynamical network biomarkers, *Scientific Reports*, **2**(342), 1-8.

13. Strogatz, S. H. *Nonlinear dynamics and chaos: with applications to physics, biology, chemistry and engineering*. (Addison-Wesley, Reading, Massachussets, 1994).

14. Liu,R. *et al.* (2012) Identifying critical transitions and their leading networks for complex diseases, *Scientific Reports*, **2**(813), 1-9.

15. Liu,R. *et al.* (2014) Identifying critical transitions of complex diseases based on a single sample, *Bioinformatics*, **30**(11), 1579-1586.

16. Scheffer, M. *et al.* Early-warning signals for critical transitions, *Nature* **461**, 53–59(2009).

17. Van Nes, E.H. & Scheffer, M. Slow recovery from perturbations as a generic indicator of a nearby catastrophic shift, *Am. Nat.* **169**, 738–747(2007).

18. Wissel, C. A universal law of the characteristic return time near thresholds, *Oecologia* **65**, 101–107(1984).

19. Becskei, A. & Serrano, L. Engineering stability in gene networks by autoregulation, *Nature* **405**, 590–593(2000).

20. Chen, L. & Aihara, K. Stability of genetic regulatory networks with time delay, *IEEE Trans. Circuits Syst. I* **49**, 602–608(2002).

21. Li, C., Chen, L. & Aihara, K. Stability of genetic networks with SUM regulatory logic: Lur'e system and LMI approach, *IEEE Trans. Circuits Syst. I* **53**,

2451–2458(2006).

22. Kloeden, P. & Platen, E. *Numerical Solution of Stochastic Differential Equations*, (Springer, 1999).

23. Rencher, A. C. *Methods of Multivariate Analysis*, (Wiley, New York, 1995).

24. Chen, P., Li, Y. The decrease of consistence probability: at the crossroad of catastrophic transition of a biological system. BMC Systems Biology, 2016, 10(2): 50.

25. Reimand, J., Arak, T. & Vilo, J. g:Profiler – a web server for functional interpretation of gene lists (2011 update), *Nucleic Acids Res* **39**, W307–315(2011).

26. Wang, J. *et al.* (2011) NOA: a novel Network Ontology Analysis method, *Nucleic Acids Res*, **39**, e87.

27. Gene cards: http://www.genecards.org/.

28. Wolfgang, S. & Werner, D. *"Phosgene" in Ullmann's Encyclopedia of Industrial Chemistry Wiley-VCH*, (Weinheim, 2002).

29. Sciuto, A. M., *et al.* (2005) Genomic analysis of murine pulmonary tissue following carbonyl chloride inhalation, *Chem. Res. Toxicol.* **18**, 1654–1660.

30. Wang, P. *et al.* Mechanism of acute lung injury due to phosgene exposition and its protection by cafeic acid phenethyl ester in the rat, *Exp. Toxicol Pathol.*, **24** (2011).

31. Saeki Y., *et al.* Ligand-specific sequential regulation of transcription factors for differentiation of MCF-7 cells, *BMC Genomics*, **20**, 545-552 (2009).

32. Fang, Y., Choi, D., Searles, R. P., & Mathers, W. D. . A time course microarray study of gene expression in the mouse lacrimal gland after acute

corneal trauma. Investigative ophthalmology and visual science, 46(2), 461-469 (2005).

33. Dartt D.A. Dysfunctional neural regulation of lacrimal gland secretion and its role in the pathogenesis of dry eye syndromes. Ocular Surface. 2:76-91 (2004).

34. Suzuki, H., Okunishi, R., Hashizume, W., Katayama, S., Ninomiya, N., Osato, N., Sato, K., Nakamura, M., Iida, J., Kanamori, M. & Hayashizaki, H. Identification of region-specific transcription factor genes in the adult mouse brain by medium-scale real-time RT-PCR, *FEBS Lett* **573**, 214–218(2004).

35. Normanno, N., Ciardiello, F., Brandt, R. & Salomon, D. S. Epidermal growth factor-related peptides in the pathogenesis of human breast cancer, *Breast Cancer Research and Treatment* **29**, 11–27(1994).

36. Huang, D. W., Sherman, B. T. & Lempicki, R.A. Systematic and integrative analysis of large gene lists using DAVID Bioinformatics Resources, *Nature Protoc* **4**, 44–57(2009).

37. Barlund, M., Monni, O., Weaver, J. D., Kauraniemi, P., Sauter, G., Heiskanen, M., Kallioniemi, O. P. & Kallioniemi, A. Cloning of BCAS3 (17q23) and BCAS4 (20q13) genes that undergo amplification, overexpression, and fusion in breast cancer, *Genes Chromosomes Cancer* **35**, 311–317(2002).

38. Casanova, J. C., Uribe, V., Badia-Careaga, C., Giovinazzo, G., Torres, M. & Sanz-Ezquerro, J. J. Apical ectodermal ridge morphogenesis in limb development is controlled by Arid3b-mediated regulation of cell movements, *Development* **138**, 1195–1205(2011).

39. Akhavantabasi, S., Sapmaz, A., Tuna, S. & Erson-Bensan, A. E. miR-125b

targets ARID3B in breast cancer cells, *Cell Struct Funct* **37**(1), 27–38(2012).

40. Kasof, G.M., Lu, J.J., Liu, D., Speer, B., Mongan, K.N., Gomes, B.C. & Lorenzi, M.V. Tumor necrosis factor-alpha induces the expression of DR6, a member of the TNF receptor family, through activation of NF-kappaB, *Oncogene.* **20**(55), 7965–7975(2001).

41. Sathyanarayana, U.G., Padar, A., Huang, C.X., Suzuki, M., Shigematsu, H., Bekele, B.N. & Gazdar, A.F. Aberrant promoter methylation and silencing of laminin-5-encoding genes in breast carcinoma, *Clin Cancer Res* **9**(17), 6389–6394(2003).

42. Koshikawa, N., Minegishi, T., Sharabi, A., Quaranta, V. & Seiki, M. Membrane-type matrix metalloproteinase-1 (MT1-MMP) is a processing enzyme for human laminin gamma 2 chain, *J Biol Chem* **280**(1), 88–93(2005).
